# Lifespan developmental invariance in memory consolidation: evidence from procedural memory

**DOI:** 10.1093/pnasnexus/pgad037

**Published:** 2023-02-08

**Authors:** Eszter Tóth-Fáber, Dezso Nemeth, Karolina Janacsek

**Affiliations:** Doctoral School of Psychology, ELTE Eötvös Loránd University, H-1064 Budapest, Hungary; Institute of Psychology, ELTE Eötvös Loránd University, H-1064 Budapest, Hungary; Brain, Memory and Language Research Group, Institute of Cognitive Neuroscience and Psychology, Research Centre for Natural Sciences, H-1117 Budapest, Hungary; Institute of Psychology, ELTE Eötvös Loránd University, H-1064 Budapest, Hungary; Brain, Memory and Language Research Group, Institute of Cognitive Neuroscience and Psychology, Research Centre for Natural Sciences, H-1117 Budapest, Hungary; Université Claude Bernard Lyon 1, CNRS, INSERM, Centre de Recherche en Neurosciences de Lyon CRNL U1028 UMR5292, F-69500 Bron, France; Institute of Psychology, ELTE Eötvös Loránd University, H-1064 Budapest, Hungary; Centre for Thinking and Learning, Institute for Lifecourse Development, School of Human Sciences, Faculty of Education, Health and Human Sciences, University of Greenwich, Old Royal Naval College, SE10 9LS London, UK

**Keywords:** consolidation, procedural memory, statistical learning, lifespan approach

## Abstract

Characterizing ontogenetic changes across the lifespan is a crucial tool in understanding neurocognitive functions. While age-related changes in learning and memory functions have been extensively characterized in the past decades, the lifespan trajectory of memory consolidation, a critical function that supports the stabilization and long-term retention of memories, is still poorly understood. Here we focus on this fundamental cognitive function and probe the consolidation of procedural memories that underlie cognitive, motor, and social skills and automatic behaviors. We used a lifespan approach: 255 participants aged between 7 and 76 years performed a well-established procedural memory task in the same experimental design across the whole sample. This task enabled us to disentangle two critical processes in the procedural domain: statistical learning and general skill learning. The former is the ability to extract and learn predictable patterns of the environment, while the latter captures a general speed-up as learning progresses due to improved visuomotor coordination and other cognitive processes, independent of acquisition of the predictable patterns. To measure the consolidation of statistical and general skill knowledge, the task was administered in two sessions with a 24-h delay between them. Here, we report successful retention of statistical knowledge with no differences across age groups. For general skill knowledge, offline improvement was observed over the delay period, and the degree of this improvement was also comparable across the age groups. Overall, our findings reveal age invariance in these two key aspects of procedural memory consolidation across the human lifespan.

**Significance statement**

Consolidation is a critical function responsible for the stabilization and long-term retention of memories. Here, we tested the consolidation of procedural memories, which underlie skills and automatic behaviors, using a lifespan approach. In contrast to the age-variant lifespan trajectory of procedural learning, our results revealed age-invariant procedural memory consolidation across the lifespan. Thus, procedural learning and consolidation seem to follow distinct developmental curves in neurotypical individuals. These findings suggest at least partially different neural underpinnings of learning versus consolidation and will likely stimulate future neuroimaging research and theory development of memory.

## Introduction

Identifying age-related changes in cognitive functions across the human lifespan is a crucial step in understanding brain development and developing more efficient diagnostic tools and interventions for developmental delays and decline in old age. Substantial research has focused on characterizing how cognitive functions change across the lifespan. The largest body of evidence comes from studies comparing the cognitive performance of typically two to four age groups. There are comparably fewer large-scale, cross-sectional, or longitudinal studies that track performance from childhood to older adulthood (i.e. across the lifespan) using the same task. These large-scale studies, however, are essential for a better understanding of cognitive changes across the lifespan as they control for a range experimental and analytical factors that cannot be controlled when lifespan trajectories are inferred based on a diverse set of individual studies. Combined evidence from these different study approaches reveals markedly distinct lifespan trajectories depending on the cognitive function of interest. For example, aspects of executive functions (1, 2), working memory (3–5), autobiographical memory (6), and episodic memory (7, 8) have been shown to follow an inverted U-shape trajectory, with continuous maturation during childhood, a peak performance in young adulthood, and a deterioration in older adulthood. In contrast, language acquisition, general skill learning, and statistical learning—the ability to extract and learn predictable patterns of the environment—seem to peak during childhood, followed by a decline in adulthood (9–11). Furthermore, certain cognitive functions might remain intact later in life as well, such as automatic processes of memory retrieval (12). While our knowledge on the age-related changes in learning and memory functions has greatly expanded in the past decades, the lifespan trajectory of memory consolidation—a critical function that is responsible for the stabilization and long-term retention of memories—is still poorly understood. Here we focus on this fundamental cognitive function and probe the consolidation of memories acquired via statistical learning using a lifespan approach.

Statistical learning is a crucial aspect of life from infancy to old age as it enables us to extract complex probabilistic regularities embedded in the environment, allowing us to adapt to our surroundings throughout the human lifespan (13–17). Through extensive practice, statistical learning contributes to the acquisition of automatic behaviors, such as skills and habits, which are rooted in procedural memory (18–21). The developmental trajectory of statistical learning has been described with three different models (22). The age-invariant model suggests no developmental changes across the lifespan (23), based on studies showing comparable learning performance in children and adults (e.g. 24), and based on results showing that statistical learning is related to brain regions that mature early, such as the striatum (23). The other two models propose that statistical learning varies as a function of age. The inverted U-shaped model suggests a gradual improvement over childhood and adolescence, with the best performance in young adulthood and a decline with aging (25). This model is supported by results finding better learning performance in young adulthood than in childhood and old adulthood (e.g. 26). Involving a large sample of participants from childhood to old adulthood, a study (25) found evidence for the inverted U-shaped model examining participants between 7 and 87 years of age. The third model, which can be referred to as “competition model,” argues for better statistical learning in childhood (under the age of 12), less effective learning in adolescence and adulthood and a decline in old adulthood (9, 10). In detail, Janacsek et al. (10) differentiate between the detection of raw probabilities and the usage of internal models. They argue that due to the yet underdeveloped internal models, children are more sensitive to raw statistical probabilities of the environment, which translates to better statistical learning performance. The development of internal models in adolescence and adulthood then leads to less reliance on raw statistical probabilities as more complex interpretations of the observed probabilities emerge. The decline in old adulthood can be explained by reduced sensitivity to raw statistical probabilities, increased rigidness of internal models, and/or a weaker connection between these two systems. Employing a lifespan approach, a study (10) investigated participants from the age of 4 to 85 years, showing better statistical learning under the age of 12. Moreover, Nemeth et al. (27) contrasted the performance of five age groups from 11 to 39 years. They showed better statistical learning in the 11- to 13-year-old group compared with the other age groups, while statistical learning was similar from the age of 14 to 39 years. A recent study by Juhasz et al. (9) examined statistical learning from the age of 7 to 85 years involving the same pool of participants as the present study. Notably, Juhasz et al. (9) focused on the trajectory of statistical learning and general skill learning (see below for details), whereas the present study focuses on the 24-h consolidation of such knowledge.

Importantly, statistical learning does not occur only during practice but also between the practices, in the so-called offline periods. Via consolidation, the initially fragile and unstable memory representations are converted into a more stable form, ensuring that they are preserved and can be retrieved later (28). Successful consolidation can be reflected by retention (i.e. no forgetting, similar performance at the end of learning and during subsequent testing) or even by offline gains (i.e. offline learning, better performance during testing than at the end of learning) (29). The consolidation of knowledge acquired via statistical learning has been tested across different time delays (e.g. from hours to days or even a year) between learning and testing, but all studies have focused on one age group at a time or contrasted performance of a couple of age groups (e.g. children vs. adults; young vs. older adults) only. The present study aims to go beyond previous research by examining consolidation of statistical knowledge across the lifespan, in a sample of participants aged between 7 and 76 years.

Despite the ample investigation on the lifespan trajectory of statistical learning, the *consolidation* of such knowledge did not receive much attention. To the best of our knowledge, no models were proposed for the lifespan trajectory of the consolidation of statistical knowledge. Considering the proposed trajectories of statistical learning, different developmental curves can be proposed for the consolidation of statistical knowledge. As described above, two age-variant trajectories have been proposed for the development of statistical learning (10, 25). It raises the question whether we can expect that the consolidation of such knowledge will also follow an age-variant trajectory. In atypical development, it has been demonstrated that learning and consolidation can show dissociation: Enhanced learning and intact consolidation has been shown in Tourette syndrome (30), whereas impaired consolidation has been shown to accompany intact learning in developmental dyslexia (31). However, it is still an open question whether learning and consolidation show a dissociation in neurotypical populations, especially across development and aging.

As described above, research on the consolidation of statistical knowledge has focused on one age group at a time or contrasted performance in a couple of age groups only. Most studies have suggested that children and adolescents can successfully retain the acquired knowledge following delays ranging from hours to one-year (30, 32–35), while others have found offline learning (i.e. improved performance) in a group of children and adolescents after a 24-h delay (31). Smalle et al. (36) investigated a related process, that is, Hebb learning in a longitudinal design with 8- to 9-year-old children and adults and tested the retention of sequences over a 4-h, 1-week, and 1-year offline delay. The results showed better consolidation in children compared with adults across all offline periods. In young and middle aged adults, knowledge of statistical regularities seems to be successfully retained, irrespective of the length of delay (e.g. 21, 37–44). In contrast, the handful of studies focusing on older adults have revealed mixed results: Some studies have suggested retention of the acquired knowledge (21), whereas others have indicated a decline over the delay period (45). Overall, based on these studies, no firm conclusions could be drawn on the consolidation of statistical knowledge across the lifespan, although retention (that is, no performance change) seems to be the most plausible outcome for most age groups, which would support an age-invariant model of the consolidation of statistical knowledge.

Since statistical learning requires repeated exposure to the same regularities (46), during this period of repeated exposure (i.e. in the learning phase), other learning processes are also engaged that could confound the measures and interpretation of statistical learning as well as its consolidation. One such learning process is called general skill learning, which refers to the faster processing of and responding to stimuli and improved visuomotor coordination as a result of practice, independent of the regularities embedded in the stimulus stream (9, 18). In the present study, we use a task design that enables us to tease apart consolidation processes specific to statistical knowledge by contrasting it to the consolidation of general skill knowledge. Unveiling the lifespan trajectory of the consolidation of statistical and general skill knowledge using a carefully controlled, identical design across age groups from childhood to older adulthood can significantly improve our understanding of how the consolidation of different types of knowledge changes across development and aging and can shed light on the age-related changes in brain plasticity supporting these functions.

With regards to the consolidation of general skill knowledge, offline improvement has been shown both in children and adults, that is, participants usually exhibited faster average reaction times after an offline period (31, 32, 34, 44, 47, 48). Nevertheless, whether the extent of offline improvement differs across development from childhood to adulthood remains unclear. In older adults, the results on the consolidation of general skill knowledge are largely mixed. Elderly participants demonstrated offline gains over a 12-h delay (45, 49), but the gain was smaller than in young adults (49). Moreover, Nemeth and Janacsek (45) did not find evidence for improvement following 24-h and 1-week delays in older adults, while young adults showed significant improvements following both delay periods. Retention but no offline improvement of general skill knowledge has been found over a 1-year delay as well, with no differences between young and older adults (21). Thus, while offline improvement of general skill knowledge may be expected in some cases (e.g. for shorter delays), overall, no conclusive pattern across studies could be revealed, especially for potential differences in the extent of this improvement from childhood to adulthood. Nonetheless, based on the previous studies, it is reasonable to expect at least some age variance for the consolidation of general skill knowledge.

To the best of our knowledge, no study has tested consolidation of statistical and/or general skill knowledge with the same experimental design across the lifespan so far. The present study fills this gap using a learning task that enables us to tease apart consolidation processes specific to statistical knowledge versus general skill knowledge in a large sample of participants aged between 7 and 76 years. By employing the same experimental design across the whole sample, our study can unveil the lifespan trajectory of the consolidation of statistical and general skill knowledge: Crucially, it can provide clear evidence for potential differences in consolidation across age groups from childhood to older adulthood as well as across knowledge types. Based on the previous empirical findings, for the lifespan trajectory of consolidation of statistical knowledge, an age-invariant trajectory can be proposed, whereas the consolidation of general skill knowledge might follow an age-variant trajectory. The findings of the present study can greatly improve our understanding of the consolidation of different types of knowledge across the human lifespan and can shed light on the age-related changes in brain plasticity supporting these functions. Unveiling the lifespan trajectory of statistical as well as general skill knowledge can also help develop a theoretical model for these processes.

## Methods

### Participants

Two hundred and seventy participants took part in the present study. They were assigned to nine age groups (*n* = 30 in each group). Fourteen participants were excluded based on outlier (above 3 SDs) performance in average response times or accuracy during the whole experiment compared with their respective age group. The developmental trajectory of statistical learning and general skill improvements in the Learning Phase of this sample are reported in Juhasz et al. (9). The present study focuses on the consolidation of statistical knowledge and general skills; these results were not reported elsewhere. For consistent age distribution, we decided to exclude one (85-year-old) participant from the oldest age group due to being outlier in terms of age. Hence, the final sample of the present study consisted of 255 participants aged between 7 and 76 years. Mean and SD for age and gender ratio for all age groups are presented in Table 1. Caregivers of underage participants completed a parental questionnaire and adults completed a self-report questionnaire regarding health-related questions. All participants had normal or corrected-to-normal vision and none of the participants had any neurological, psychiatric, or neurodevelopmental disorder. Adult participants gave informed written consent, whereas caregivers of underage participants provided informed written consent and children and adolescents provided verbal consent to participate in the study before enrollment. Participants received no financial compensation for participation. All experimental procedures were approved by University Research Ethics Committee, and were conducted in accordance with the Declaration of Helsinki.

**Table 1.** Demographic data (mean and SD for age and gender ratio) for all age groups.

Group	Age	Gender
7–8 years old (*n* = 26)	7.92 (0.27)	13 M/13 F
9–10 years old (*n* = 28)	9.79 (0.42)	13 M/15 F
11–13 years old (*n* = 30)	12.10 (0.61)	13 M/17 F
14–15 years old (*n* = 30)	14.55 (0.57)	13 M/17 F
16–17 years old (*n* = 30)	16.56 (0.54)	13 M/17 F
18–29 years old (*n* = 30)	21.64 (2.93)	12 M/18 F
30–44 years old (*n* = 30)	36.67 (3.81)	12 M/18 F
45–60 years old (*n* = 26)	51.65 (4.46)	6 M/20 F
61–76 years old (*n* = 25)	65.28 (4.47)	5 M/20 F

### Task

**Fig. 1.**
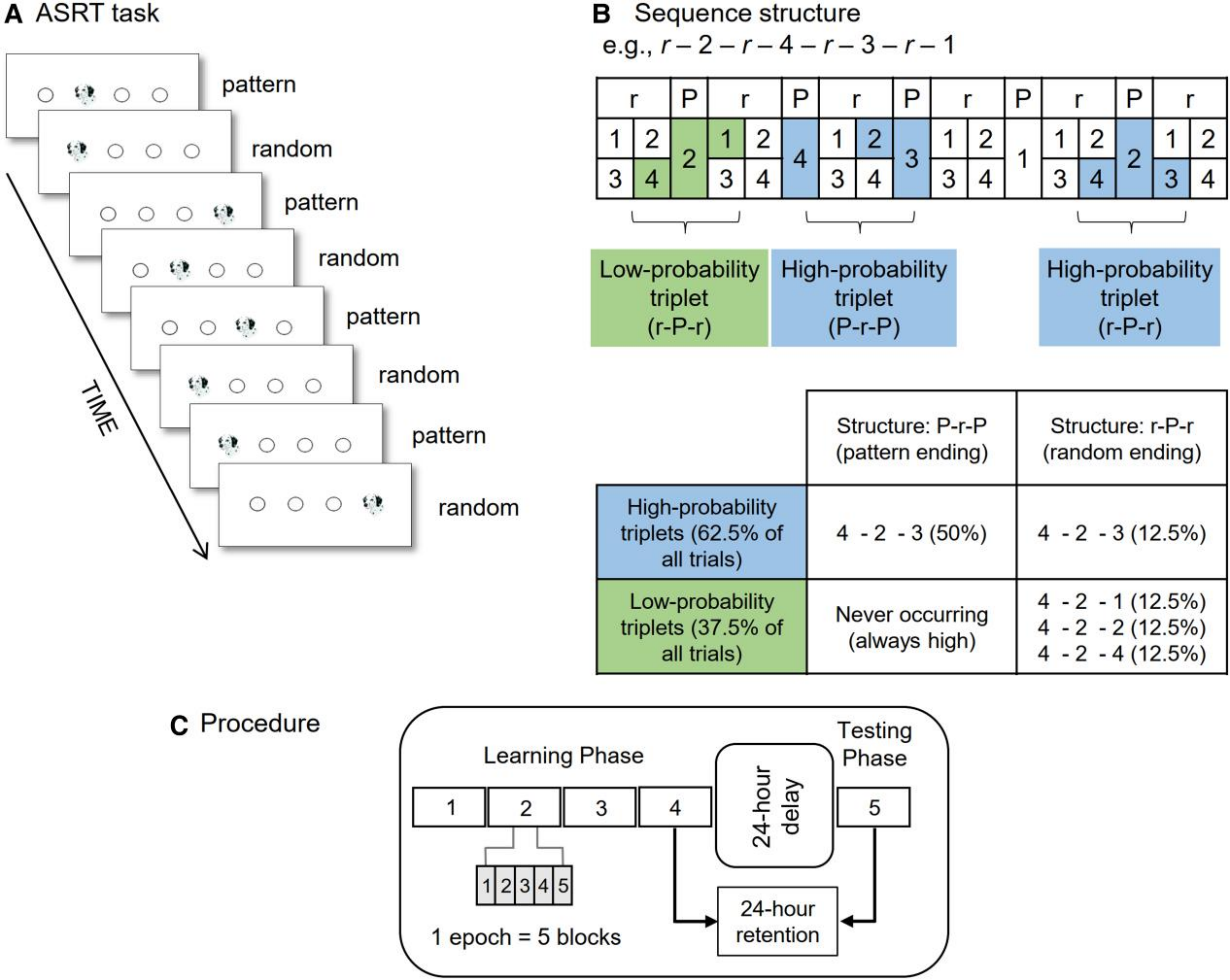
The Alternating Serial Reaction Time (ASRT) task. (A) Pattern and random trials were presented in an alternating fashion; the trial types were indistinguishable on the surface level: a picture of a dog's head served as stimuli in all trials. The alternating sequence was coded by the location of stimuli. In pattern trials, the location of stimuli was predetermined, and occurred in the same order throughout the experiment. In random trials, randomly chosen locations out of the four possible ones were presented. (B) An example of the sequence structure. Numbers indicate the predetermined stimulus locations in pattern trials, and *r*s indicate randomly selected locations out of the four possible ones. Due to the alternating sequence, some runs of three consecutive trials (triplets) were more probable than others, referred to as high-probability (green shading) and low-probability triplets (blue shading), respectively. Since high-probability triplets could occur as pattern-ending triplets (50% of all trials) and by chance as random-ending triplets (12.5% of all trials), these triplets constituted 62.5% of all trials. Low-probability triplets constituted the remaining 37.5% of the trials; these were all random-ending triplets. Note that triplets were identified using a moving window throughout the stimulus stream: each trial was categorized as the third element of a high- or a low-probability triplet; the same trial then served as the middle and the first element for the categorization of the following triplets. (C) Experimental procedure. The experiment consisted of two sessions. The Learning Phase was composed of four epochs (each epoch contained five blocks with 85 trials in each block). The Testing Phase consisting of one epoch was administered 24-h later. Figs. 1A and 1B are adapted from Nemeth et al. (27) and Zavecz et al. (52), and Fig. 1C is adapted from Kóbor et al. (41).

The Alternating Serial Reaction Time (ASRT) task was used to assess statistical learning and consolidation (49, 50). In this task, four horizontally arranged empty circles are presented on the screen and a stimulus (a dog's head) appeared in one of the circles (51). Participants were instructed to press a corresponding key (Z, C, B, or M on a QWERTY keyboard) as quickly and accurately as they could when the stimulus occurred using their index and middle fingers. After the correct response of the participant, the next stimulus appeared 120 ms later. Unbeknownst to the participants, the presentation of stimuli followed an eight-element sequence, within which pattern (P) and random (r) trials alternated with each other (e.g. 2-r*-*4-r*-*3-r*-*1-r; where numbers indicate the four locations on the screen from left to right, and r denote a randomly chosen location out of the four possible ones; see Fig. 1).

Due to this alternating sequence, some runs of three consecutive trials (triplets) were more probable than others. In the example sequence 2-r*-*4-r*-*3-r*-*1-r, triplets 2-X-4, 4-X-3, 3-X-1, and 1-X-2 (where X indicates the middle element of the triplet) occurred with a higher probability because they were presented in every sequence repetition (P-r-P) and could also be formed by chance (r-P-r, see Fig. 1B). Note that here, we use X to indicate the middle element of the triplet because, for example, 4-X-3 (e.g. 4-2-3 in Fig. 1B) can appear both as a P-r-P structure (where the first and last element of the triplet belong to the predetermined pattern) and as a r-P-r structure (where the first and last elements are random, and the middle element is part of the predetermined pattern). In contrast, triplets 2-X-1 and 3-X-2 occurred with a lower probability since they could only be formed by chance (that is, their structure could only be r-P-r). The former triplet types are referred to as high-probability triplets and the latter ones as low-probability triplets. Overall in the task, high-probability triplets were five times more probable than the low-probability ones (27, 41). Note that triplets were identified using a moving window throughout the stimulus stream. Thus, each trial was categorized as the third element of a high- or a low-probability triplet, and this categorization was used in our analyses; the same trial then served as the middle and the first element for the categorization of the following triplets.

The ASRT task enables us to separate statistical learning from general skill improvements. Statistical learning is defined as faster and more accurate responses to high-probability elements than to low-probability ones (50). In contrast, general skill improvements refer to average speed-up and changes in accuracy which are independent of the probabilities of events. These improvements reflect more efficient visuomotor and motor–motor coordination due to practice (9, 18).

### Procedure

The ASRT task was presented in blocks. One block consisted of 85 trials: each block started with 5 random practice trials followed by the 8-element sequence repeated 10 times. After each block, participants received feedback about their general performance, that is, about their average RTs and accuracy. The ASRT task was administered in 2 sessions with a 24-h delay between them (see Fig. 1C). The Learning Phase consisted of 20 blocks. The Testing Phase contained five blocks.

For the alternating sequence, there were 24 permutations of the four possible spatial positions for the predetermined order of pattern trials. However, because of the continuous presentation of the stimuli, for instance, the sequences 2-r-1-r-3-r-4, 1-r-3-r-4-r-2, 3-r-4-r-2-r-1, and 4-r-2-r-1-r-3 were considered identical as they consisted of the same triplets. Consequently, there were six *unique* sequence permutations: 1-r-2-r-3-r-4-r, 1-r-2-r-4-r-3-r, 1-r-3-r-2-r-4-r, 1-r-3-r-4-r-2-r, 1-r-4-r-2-r-3-r, and 1-r-4-r-3-r-2-r. One of these unique permutations was selected for each participant in a pseudorandom manner. For a given participant, the sequence permutation remained the same over the Learning and Testing Phases.

In our study, participants were not informed about the underlying probability structure of the sequence, and they did not even know that they were in a learning situation. Nevertheless, potentially emerged explicit knowledge about the structure was probed by a questionnaire at the end of the Testing Phase (39, 49). None of the participants reported noticing the sequence in the task. Thus an implicit, nonconscious form of learning was tested (53–55). This is in line with previous studies showing that participants remain unaware of the sequence even after extended practice, or when more sensitive recognition tests are used to assess explicit knowledge (39, 56).

### Statistical analysis

Statistical analysis was based on previous studies (41, 49, 57); to facilitate data processing, epochs of five blocks were analyzed instead of single blocks (e.g. Blocks 1–5 corresponded to Epoch 1, Blocks 6–10 to Epoch 2, and so on). The Learning Phase consisted of four epochs, while the Testing Phase consisted of one epoch. Similarly to previous studies, two types of low-probability triplets, repetitions (e.g. 222, 333) and trills (e.g. 212, 343), were eliminated because people often show preexisting response tendencies to them (56, 58). By eliminating these triplets, we could ensure that any high- versus low-probability differences were due to statistical learning and not to preexisting tendencies. We calculated mean accuracy and median RTs (for correct responses) for each participant and each epoch, separately for high- and low-probability triplets. The mean accuracy was 95.19% (SD = 0.03%) in the Learning Phase of the task. Since high accuracy scores and the relatively low variance in samples of neurotypical participants can hinder the detection of learning (59), we considered RTs to be a more appropriate measure of performance in the ASRT task. Therefore, we use RTs as our primary measures in this paper. Statistical learning scores were calculated as the difference in RTs between high- and low-probability triplets (i.e. RTs for low-probability triplets minus RTs for high-probability triplets). Higher scores indicated better learning/memory performance. General skill knowledge was defined as a general decrease in median RTs during practice (i.e. participants became faster throughout the task), irrespective of triplet types. Median RTs were calculated separately for each epoch in each phase.

To evaluate statistical learning, we conducted repeated measures ANOVAs by contrasting statistical learning scores across the Learning Phase. To test general skill learning, we contrasted median RTs across the Learning Phase using repeated measures ANOVAs. As the main goal of the present paper is to investigate the consolidation of statistical and general skill knowledge, we only briefly report the results on learning in the main text and report the exact statistics in the Supplementary materials. To evaluate the consolidation of the acquired statistical knowledge, we conducted ANOVAs by contrasting statistical learning scores of the last epoch of the Learning Phase with those of the first epoch of Testing Phase. To evaluate the consolidation of general skill knowledge, we conducted repeated measures ANOVAs by contrasting median RTs of the last epoch of the Learning Phase with those of the first epoch of the Testing Phase. Greenhouse–Geisser epsilon (*ε*) correction was used when necessary. Original d*f* values and corrected, two-tailed *P*-values (if applicable) are reported together with partial eta-squared (*η_p_*^2^) as the measure of effect size.

As children and older adults are typically respond with slower RTs overall (e.g. 9), we conducted additional ANOVAs on standardized RTs. To control for the effect of average RT differences across age groups on learning and consolidation of knowledge, we employed two different ways of standardization: (i) ratio scores and (ii) log-transformed RT data. For calculating ratio scores, we transformed the data in the following way. We divided each participants’ raw RT values of each trial type and each epoch by their own median RT in the first epoch of the task (for a similar approach, see 9, 35, 43, 60). This way, participants’ performance was around 1 at the beginning of the task and changed as the task progressed. We then calculated standardized learning and memory scores by subtracting standardized RTs for high-probability triplets from standardized RTs for low-probability triplets. Higher standardized scores indicated better learning/memory. General skill knowledge scores were standardized in an identical way: Each participants’ median RTs in Epoch 4 and Epoch 5 were divided by their median RT in the first epoch of the task. For log-transformed RT data, we applied a log *N* transformation on the trial-based raw RT data. Then, we computed the mean of log-transformed RTs for each trial type and each epoch, separately for each participant. Log-transformed statistical knowledge scores were calculated by subtracting log-transformed RTs for high-probability triplets from log-transformed RTs for low-probability triplets. Log-transformed general skill knowledge was calculated for each epoch using the mean of trial-based log-transformed RT data. For the sake of brevity, we only refer to the results of these ANOVAs in the main text, where they are relevant in comparison with the results of raw RTs, and we report the exact statistics in the Supplementary Materials.

Moreover, to explore consolidation in more detail, we fitted curves to the data of the Learning Phase and used the fitted parameters to predict statistical learning scores and general skill performance in the Testing Phase (see 61–63). A linear function was fitted to the block-wise statistical learning scores, and a power function was fitted to the block-wise general skill learning scores. Since some participants acquire statistical regularities quickly, showing high statistical learning early in the task, then maintaining their performance throughout the task, the slope of their learning trajectory is near zero. This leads to a low *R*^2^ value even when a linear function fits the data well. Residual standard errors (RSEs) are independent of the slope; therefore, they are better goodness-of-fit estimates in these cases. Hence, we report RSEs instead of *R*^2^ values, both for the statistical learning and general skill learning scores, for comparability. Smaller RSEs indicate better fit in both cases.

Importantly, in previous studies that used curve fitting on learning data, a performance improvement (i.e. offline learning) was typically expected after an offline delay (61–63). In these cases, using fitted parameters from a power or a linear function has been an appropriate approach to predict and test future performance (61). Therefore, using curve fitting to test offline changes can work well for general skill learning in our study because offline learning is expected following the 24-h delay. However, this approach may be less ideal for statistical learning as measured by the ASRT task because maintenance of performance (i.e. retention) may be expected instead of offline learning (e.g. 37, 39, 41). Moreover, differences in variance across age groups can create additional challenges when curve fitting is used to test age-related differences in learning and consolidation: As variance can influence how well a function fits the data and how reliable the predicted performance is, differences in variance can hinder the comparability of predicted performance across age groups. Nevertheless, we report the curve fitting results to provide a more detailed picture of the consolidation of statistical and general skill knowledge across the lifespan using multiple approaches.

In conjunction with the frequentist analyses, we performed Bayesian mixed-design ANOVAs and Bayesian paired-samples *t*-tests for the relevant comparisons. Bayesian mixed-design ANOVAs were run on the memory scores to test which factors determine performance. Here, we present Bayesian model averaging and the exclusion Bayes factor (BF_exclusion_). BF_exclusion_ values quantify the change from prior to posterior odds and can be interpreted as the evidence in the data for excluding a given predictor from the model. Thus, values below 1 support the inclusion and values above 1 the exclusion of the given factor. Cauchy prior distribution was used for the ANOVA with a fixed-effects scale factor of *r* = 0.5, and a random-effects scale factor of *r* = 1. Moreover, we ran Bayesian paired-samples *t*-tests for comparing performance between the end of the Learning Phase and the beginning of the Testing Phase, separately for each age group. Bayes factor (BF) was computed to assess the amount of evidence for the null-hypothesis of no offline change. The BF is a statistical technique that helps conclude whether the collected data favors the null-hypothesis (i.e. evidence for no difference between groups or variables) or the alternative hypothesis (i.e. evidence for differences); thus, the BF could be considered as a weight of evidence provided by the data (64). One of the main benefits of calculating the BF is that for nonsignificant comparisons we can use the BF to conclude that the acquired evidence supports *H*_0_ rather than *H*_1_ (65–67). BFs were calculated using JASP version 0.14 (68). Here we report BF_01_ values where greater values support the null-hypothesis (no difference) over the alternative hypothesis. According to Wagenmakers et al. (64), BF_01_ values between 1 and 3 indicate anecdotal evidence, values between 3 and 10 indicate substantial evidence and values larger than 10 indicate strong evidence for H_0_. Values between 1 and 1/3 suggest anecdotal evidence, values between 1/3 and 1/10 indicate substantial evidence, and values below 1/10 indicate strong evidence for H_1_. Values around 1 do not support either hypothesis.

## Results

### Are there age-related differences in the consolidation of statistical knowledge?

Before testing the age-related differences in consolidation of statistical knowledge, we tested the potential age-related differences in statistical learning. Analysis on raw RT data in the Learning Phase showed better learning under the age of 13, whereas analysis on ratio scores revealed comparable learning from childhood to young adulthood, followed by decreased learning from the age of 30. We present the exact statistics in the Supplementary Materials.

**Fig. 2.**
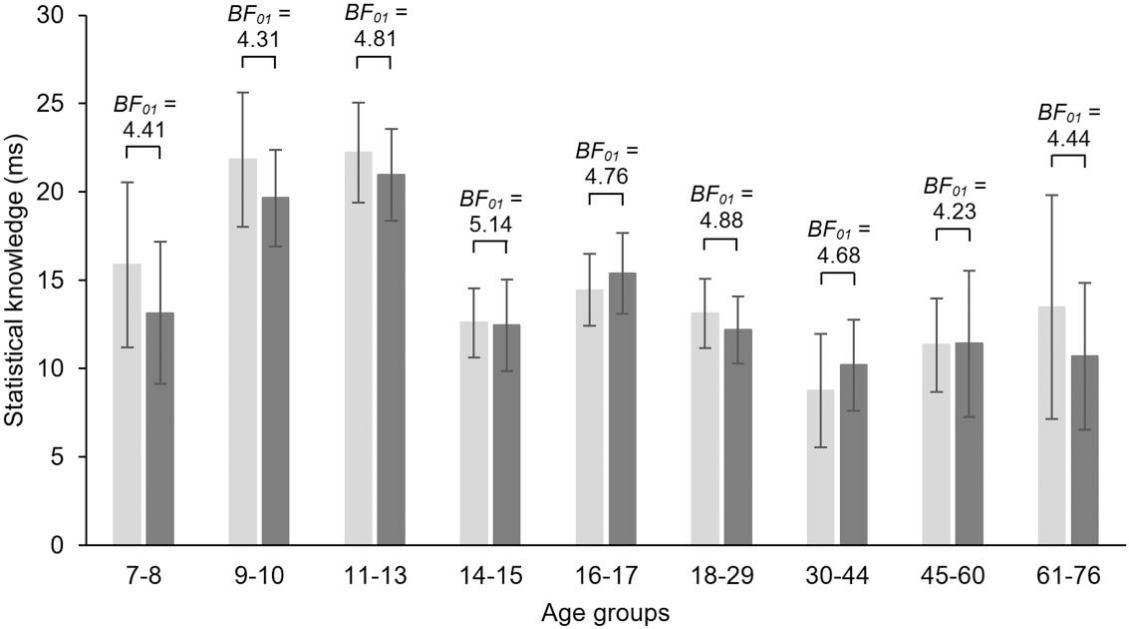
Consolidation of statistical knowledge over the 24-h offline period across age groups. RT statistical learning scores for the last epoch of the Learning Phase (Epoch 4, light gray bars) were contrasted with those for the first epoch of the Testing Phase (Epoch 5, dark gray bars). BF_01_ values were obtained by paired-samples *t*-tests for this contrast separately for each age group. All reported BF_01_ values indicate substantial evidence for the null-hypothesis (BF_01_ > 3), providing evidence for comparable knowledge in Epoch 4 and Epoch 5 in each age group. Error bars denote the standard error of mean.

To test 24-h consolidation of the acquired statistical knowledge, we contrasted statistical learning scores computed for the last epoch of the Learning Phase (Epoch 4) with the learning scores computed for the first epoch of the Testing Phase (Epoch 5) and submitted these scores to a mixed-design ANOVA with EPOCH (Epoch 4 vs. Epoch 5) as a within-subject factor and AGE GROUP as a between-subjects factor. The ANOVA revealed overall significant statistical knowledge (main effect of INTERCEPT: *F*(1, 246) = 309.24, *P* < 0.001, *η_p_*^2^ = 0.56) and significant differences in overall learning across age groups (main effect of AGE GROUP: *F*(8, 246) = 2.91, *P* = 0.004, *η_p_*^2^ = 0.09). Importantly, statistical knowledge appears to be retained over the 24-h delay period with no significant change between the end of the Learning Phase and the Testing Phase (main effect of EPOCH: *F*(1, 246) = 0.39, *P* = 0.53, *η_p_*^2^ = 0.002). Moreover, no age group differences emerged in the retention of the statistical knowledge (non-significant EPOCH × AGE GROUP interaction: *F*(8, 246) = 0.14, *P* = 0.997, *η_p_*^2^ = 0.005; all *P* > 0.52): this suggests that all age groups retained the acquired knowledge over the 24-h delay period (Figs. 2 and S1). The analysis of effects of the Bayesian mixed-design ANOVA showed that the main effect of EPOCH and the EPOCH × AGE GROUP interaction should be excluded from the model (see Table 2, and for model comparisons, see Table S1), corroborating the findings of the frequentist ANOVA.

**Table 2.** Analysis of effects of Bayesian ANOVA for consolidation of statistical knowledge.

Effects	*P* (incl)	*P* (incl|data)	BF_exclusion_
Epoch	0.400	0.104	8.608
Age group	0.400	0.800	0.250
Epoch × age group	0.200	3.382e−4	246.000

The Effects column denotes the main effects and interaction. The *P*(incl) column indicates the prior inclusion probability and the *P*(incl|data) denotes the posterior inclusion probability. The BF_exclusion_ column shows the exclusion Bayes factors. BF_exclusion_ values below 1 support the inclusion and values above 1 the exclusion of the given factor.

To rule out the possible confounding effect of different average RTs across age groups on these results, we employed two ways of standardization, and we conducted two ANOVAs for ratio scores and log-transformed RT data, respectively (for details on the standardization process, see the Statistical analysis section). We submitted the standardized statistical learning scores to mixed-design ANOVAs with EPOCH (Epoch 4 vs. Epoch 5) as a within-subject factor and AGE GROUP as a between-subjects factor. The ANOVAs revealed identical results to the ANOVA computed on raw RT scores (for the exact statistics, see Table S7 and the accompanying text). The Bayesian mixed-design ANOVAs on the standardized learning scores also supported these findings (for details, see Tables S3–S4 and S8–S9), confirming no change in learning scores over the 24-h delay period and no differences in this pattern across age groups.

To explore consolidation in more detail, we used a linear function to predict performance in the Testing Phase (for details, see the Statistical analysis section). RSEs were calculated separately for the age groups, and they were between 3.80 and 19.17 (*M* = 8.41), suggesting a generally good fit to the data. A difference score was calculated by subtracting the predicted statistical learning scores from the observed statistical learning scores. We submitted the difference scores to a mixed-design ANOVA with BLOCK (1–5) as a within-subject factor and AGE GROUP as a between-subjects factor. Importantly, we found no significant age-related effect in this ANOVA: the difference score between the predicted and observed statistical learning scores was comparable across the age groups. Bayesian ANOVA further supported these results. For the exact statistics, see Tables S13–S15.

### Are there age-related differences in the consolidation of general skill knowledge?

Similar to statistical learning, before comparing the age groups on the consolidation of general skill knowledge, we first tested the age-related differences in general skill learning. Analysis on raw RT data in the Learning Phase revealed highest general skill learning performance in the youngest age group. For details, see the exact statistics in the Supplementary Materials.

**Fig. 3.**
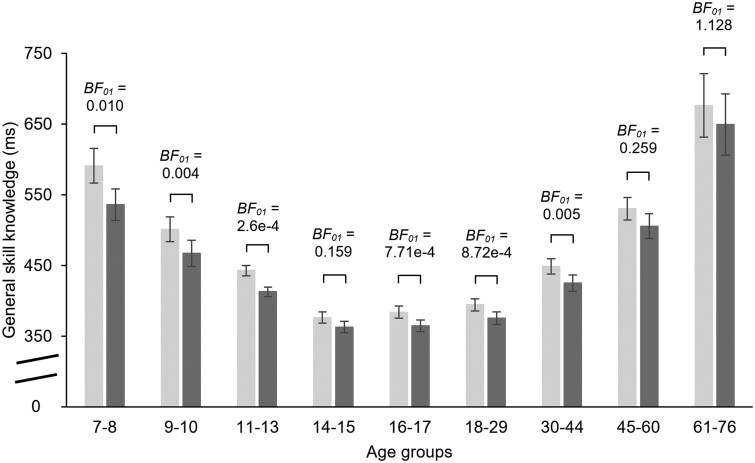
Consolidation of general skill knowledge over the 24-h offline period across age groups. Average RT values for the last epoch of the Learning Phase (Epoch 4, light gray bars) were contrasted with those for the first epoch of the Testing Phase (Epoch 5, dark gray bars). BF_01_ values were obtained by paired-samples *t*-tests for this contrast separately for each age group. BF_01_ values for all age groups except for the 61- to 76-year olds indicate substantial evidence for the alternative hypothesis (BF_01_ < 0.33) providing evidence for offline learning over the 24-h delay. BF_01_ value obtained for the 61- to 76-year olds could not provide evidence for either the null or the alternative hypotheses. Error bars denote the standard error of mean.

We tested the consolidation of general skill knowledge (defined as median RTs changes) over the delay period with a mixed-design ANOVA on median RTs (i.e. RTs irrespective of the probabilities of events) with EPOCH (Epoch 4 vs. Epoch 5) as a within-subject factor and AGE GROUP as a between-subjects factor. Our analysis found that the median RTs significantly decreased over the 24-h delay (main effect of EPOCH: *F*(1, 246) = 107.92, *P* < 0.001, *η_p_*^2^ = 0.31): participants responded faster in the Testing Phase compared with the end of the Learning Phase (significant speed-up in all age groups: all *P* < 0.014, except for the 14- to 15-year-old group, where *P* = 0.080, Fig. 3). The amount of speed-up over the delay period, however, was not uniform across the age groups (EPOCH × AGE GROUP interaction: *F*(8, 246) = 2.26, *P* = 0.02, *η_p_*^2^ = 0.07). A follow-up ANOVA on the offline change score (i.e. RTs in Epoch 4 minus RTs in Epoch 5) showed that the 7- to 8-year olds exhibited the greatest speed-up over the delay, significantly differing from the speed-up of almost all other age groups (*P* < 0.026; 7- to 8-year-old vs. 9- to 10-year-old groups: *P* = 0.068; Fig. S2). The other age groups’ median RT changes over the delay period were not significantly different from one another (all *P* > 0.062). Bayesian mixed-design ANOVA also supported the inclusion of the main effect of EPOCH and the EPOCH × AGE GROUP interaction (Table 3, and for model comparisons, see Table S2).

**Table 3.** Analysis of effects of Bayesian ANOVA for consolidation of general skill knowledge.

Effects	*P*(incl)	*P*(incl|data)	BF_exclusion_
Epoch	0.400	0.548	1.067e−17
Age group	0.400	0.548	5.163e−29
Epoch × age group	0.200	0.452	1.214

The Effects column denotes the main effects and interaction. The *P*(incl) column indicates the prior inclusion probability and the *P*(incl|data) denotes the posterior inclusion probability. The BF_exclusion_ column shows the exclusion Bayes factors. BF_exclusion_ values below 1 support the inclusion and values above 1 the exclusion of the given factor.

Similarly to the statistical learning scores, we ran two ANOVAs on standardized RTs as well, one for ratio scores and one for log-transformed RT data (for details on the standardization process, see the Statistical analysis section). Both ANOVAs revealed a significant RT speed-up over the delay period; however, in contrast to the ANOVA on raw RT scores, the age groups did not differ from each other in the amount of speed-up either concerning ratio scores or log-transformed RT data (for the exact statistics, see Table S10, Fig. S3 and the accompanying text). The Bayesian mixed-design ANOVAs also supported the inclusion of the main effect of EPOCH and the exclusion of the EPOCH × AGE GROUP interaction, suggesting a uniform speed-up over the delay period across the age groups (for details, see Tables S5–S6 and S11–S12). These results suggest that the group differences observed in the raw average RT analyses above were largely driven by some age groups being on average slower in the task than other groups; controlling for this confound eliminated the group differences in the consolidation of general skill knowledge over the delay period.

Similarly to the statistical learning scores, we further examined the magnitude of offline gains and possible age-related differences by predicting future general skill performance in the Testing Phase with a power law function (for details, see the Statistical analysis section). RSEs were calculated separately for the age groups, and they were between 4.32 and 20.54 (*M* = 10.53), suggesting a generally good fit to the data. A difference score was calculated by subtracting the predicted RT data from the observed RT data. We submitted the difference scores to a mixed-design ANOVA with BLOCK (1–5) as a within-subject factor and AGE GROUP as a between-subjects factor. We found no significant age-related effect: the difference between the predicted and observed performance was comparable across the age groups. Bayesian ANOVA further supported these results. For the exact statistics, see Tables S16–S18.

### Testing possible confounds influencing the consolidation of statistical and general skill knowledge

To test the possible confounding effect of averaging over the last five blocks of the Learning Phase and the first five blocks of the Testing Phase (61), we contrasted performance in the last block of the Learning Phase (Block 20) and in the first block of the Testing Phase (Block 21). The analysis revealed that some degree of forgetting might be present as statistical learning score in Block 21 was marginally lower than that in Block 20. Bayesian analysis was not conclusive, the BF was around 1, not supporting either the null or the alternative hypothesis. Crucially, age-related differences were not detected either with the frequentist or with the Bayesian analysis. For the exact statistics, see Tables S19 and S20 and the accompanying text.

It is important to note that the analysis of block-wise data in the ASRT task should be interpreted carefully due to the relatively low number of trials. Specifically, statistical learning scores are calculated as difference scores between the high- and low-probability trials after excluding the first five random practice trials at the beginning of the block, erroneous responses as well as trills and repetitions from the 85 trials that are presented in a block. Hence, aggregated (mostly epoch-level) data have been used to characterize learning in the ASRT task since its inception because it enables tracking the trajectory of learning while simultaneously decreasing the effect of noise in the learning scores to an acceptable level (39, 50, 69).

Moreover, to test whether practice-dependent changes influenced the offline learning of general skill knowledge over the 24-h offline delay, we compared performance in the last block of the learning phase (Block 20) and the first block of the Testing Phase (Block 21). Overall, participants responded faster in Block 21 compared with Block 20, with similar speed-up across the age groups, and the amount of speed-up was comparable across the age groups. This suggests that the offline learning of general skill knowledge over the delay period was not due to further practice-dependent changes in the Testing Phase. For the exact statistics of this analysis, see Table S21 and the accompanying text.

We also tested whether offline learning of general skill knowledge in terms of RTs could be influenced by decreased accuracy over the offline period. On the group level, mean accuracy scores increased over the offline delay, but the level of improvement was not comparable across the age groups. Significant offline learning was only detectable in the 7- to 8-year-old and 11- to 13-year-old groups. These results suggest that, in terms of accuracy scores, none of the age groups showed forgetting, therefore, offline learning in terms of RTs cannot be explained by a decreased accuracy over the offline period. For exact statistics, see Supplementary Materials.

## Discussion

The present study examined the 24-h consolidation of statistical and general skill knowledge in a large sample of participants between the age of 7 and 76 years using the same experimental design across the sample. Based on statistical learning scores computed from raw RT data, we showed retained statistical knowledge in all age groups. Analyses on standardized RT data and Bayesian analyses (both on raw RTs and standardized RTs) further corroborated these results. As for general skill knowledge, while the analyses on raw RT data suggested offline improvement that was the greatest in the 7- to 8-year olds, results on standardized RT data revealed offline gains in all age groups with a uniform speed-up across the sample. Bayesian analyses of general skill consolidation also confirmed this uniform speed-up.

Our results on age-invariance in the retention of statistical knowledge are in line with prior smaller scale studies that focused on one age group only or contrasted performance in a few age groups. Specifically, in groups of children and adolescents, successful retention of statistical regularities has been shown following a 16-h (30, 32), 24-h (34), 3-day (33), and even a 1-year offline period (35). Offline learning has also been found over a 24-h delay in a group of 9- to 13-year-old participants (31). Successfully retained knowledge had been consistently demonstrated in young and middle adulthood as well over various offline delays ranging from hours to even 1 year (e.g. 21, 38, 40, 41, 44).

However, differences in consolidation between children and adults have been suggested in specific areas, such as the contribution of sleep to consolidation. Fischer et al. (70) compared the consolidation of statistical knowledge in 7- to 11-year-old children and young adults after offline periods of overnight sleep or daytime wakefulness. Adults showed better retention of statistical knowledge following sleep compared with the wakeful offline period, whereas children showed an opposite pattern with better retention after daytime wakefulness than after overnight sleep. Notably, children showed overall higher learning than adults and this difference in the pre-sleep performance level could have strongly confounded the results on consolidation (71). Our study was not designed to test the effect of sleep on consolidation across the lifespan: the 24-h delay employed in our study included both periods of overnight sleep and daytime wakefulness. In theory, an opposite pattern such as the one observed in the study of Fischer et al. (70) could have resulted in overall similar retention performance of statistical knowledge across age groups. Importantly, we chose the 24-h delay because that could provide a clearer picture of everyday functioning of individuals as it incorporates both the effects of sleep and daytime wakefulness. Overall, consolidation of statistical knowledge seems to be similar across childhood and adulthood, and previously found developmental differences might be explained by confounding factors, such as differences in sleep.

Previous studies investigating the consolidation of statistical knowledge in aging showed somewhat mixed results. Most studies suggested intact consolidation of statistical knowledge in elderly adults. Comparable retention has been shown in younger and older adults after a 12-h offline period, irrespective of whether that period included overnight sleep or daytime wakefulness (49). Retained knowledge has also been found following a 1-year delay (21). In contrast, Nemeth and Janacsek (45) have found forgetting of statistical knowledge in older adults compared with successful retention in young adults, irrespective of the length of the offline delay (12-h, 24-h, or 1-week). Our results align with the former studies, providing substantial evidence for retained statistical knowledge over the 24-h delay in older adults, as indicated by the Bayesian analyses (BF_01_ = 4.44). The mixed findings across studies could be attributed to differences in experimental designs, for example, different lengths of the learning period could lead to a varying degree of fatigue, which can potentially affect consolidation. Future studies may systematically test how such differences affect consolidation across age groups.

During learning, we extract the regularities from the environment and encode them into initially fragile memory representations. During consolidation, the recently acquired representations undergo a progressive stabilization, creating long-term memory representations (28). Although these two processes are intertwined and depend on each other, considering the age-variant lifespan trajectory of statistical learning based on previous studies (9, 10, 25) and the age-invariant lifespan trajectory of consolidation of statistical knowledge based on the present study, we can conclude that these two processes show a dissociation. Similar results have been demonstrated before in atypical development. In Tourette syndrome, intact consolidation has been shown to accompany enhanced learning (30), whereas intact learning and impaired consolidation have been found in developmental dyslexia (31). Our results are in line with these prior ones as we showed a dissociation between learning and consolidation of statistical knowledge in a neurotypical population: while learning varied with the function of age, consolidation did not. This suggests that, at least partially, distinct mechanisms and neural networks underlie the acquisition and consolidation of statistical knowledge across the lifespan.

To the best of our knowledge, no theoretical framework has been proposed for the lifespan trajectory of consolidation of statistical knowledge. Here, we found comparable retention of statistical knowledge in all ages from 7 to 76 years, which would suggest an age-invariance model of consolidation of such knowledge. Based on the present behavioral results, we can also make assumptions for the neural networks that underlie the consolidation of statistical knowledge, at least when measured with the ASRT task. As mentioned in the introduction, in their model for statistical learning, Janacsek et al. (10) proposed a shift from detecting raw probabilities to relying more on internal interpretations of events, which then leads to decreased statistical learning performance in adults compared with children under the age of 12. On the neurobiological level, the competition model (10) related this shift to the protracted maturation of the hippocampus and prefrontal cortex. While these brain regions have been suggested to underlie the development of internal models, basal ganglia, particularly the striatum, have been linked to the detection of raw probabilities. Albouy et al. (72) proposed a model for a related process, that is, motor sequence learning and consolidation. According to this model, in healthy young adults, during the acquisition of a motor sequence, the hippocampus and the striatum show an antagonistic dynamic, which is presumably mediated by the prefrontal cortex. In contrast, during consolidation and retest, instead of a competitive dynamic, the striatum and the hippocampus seem to function cooperatively. According to Albouy et al. (72), striatal activity supports time-dependent maintenance in performance (i.e. retention), whereas the hippocampus supports sleep-dependent enhancement in performance (i.e. offline learning), at least in young adults. In line with this dissociation, Schapiro et al. (73) also showed the involvement of the hippocampus in the sleep-dependent consolidation of a motor sequence in middle aged adults. Converging our behavioral results and the model of Albouy et al. (72), we can speculate that consolidation of statistical knowledge was likely more reliant on the striatum and its circuits as we found retention of statistical knowledge in all age groups. This would be consistent with the notion that the consolidation of statistical knowledge, at least when measured with the ASRT task, is independent of sleep (e.g. 39, 49). It is important to note that in the present study, we employed a visuomotor task involving temporally distributed non-adjacent statistical regularities; therefore, the conclusion of age-invariance is restricted to these regularities. Considering other, related processes, such as motor (sequence) learning, usually measured with deterministic SRT and finger tapping tasks, a different trajectory might emerge. As stated above, the consolidation of motor (sequence) knowledge appears to be sleep dependent, resulting in offline gains over the delay, due to the involvement of the hippocampus (74). Due to sleep dependency and the underlying neural circuits (i.e. the hippocampus showing protracted development, 75), age variance may be expected for the consolidation of motor (sequence) knowledge. Indeed, empirical evidence supports this notion (76–78, but see 61, 79). In contrast, the time-dependent consolidation of statistical knowledge seems to be age invariant, potentially due to the greater reliance on the striatum that matures early in development. To sum up, the cooperation of the striatum and the hippocampus could be responsible for the consolidation of the acquired knowledge and based on our results, the striatum may be more prominent in this interaction when consolidation is independent of sleep, as is the case for the statistical knowledge tested in the present study. Nevertheless, it is important to note that this is highly tentative and further neuroimaging studies are needed to corroborate it.

Previous development and aging studies have highlighted the importance of baseline RT differences across the lifespan. It is well established that children and older adults show slower RTs compared with young adults (e.g. 9, 10). These differences can confound performance as children and older adults have more room to improve, meaning that as their baseline RTs are slower, they can demonstrate higher gains in learning. Juhasz et al. (9) focused on general skill learning in the sample used in the present study. According to their results, general skill learning is heightened in childhood, but this could not be explained by the “more room to improve” concept. Superior general skill learning in 7- to 8-year olds persisted across different analysis approaches which controlled for the baseline speed differences. Our results on raw RT data showed that 7- to 8-year olds are also superior in the consolidation of general skill knowledge as they exhibited greater offline improvement than the other age groups. Importantly, however, this could not be confirmed by the analyses of standardized RT data where we controlled for the average speed differences across the age groups. Hence, the greater offline speed-up compared with other age groups seen on raw RT data is possibly due to the generally slower responses in the 7- to 8-year olds. Thus, while general skill learning seems to be age variant with heightened learning in childhood (9), consolidation of such knowledge seems to be age invariant with similar offline improvement in all age groups, based on the results of the current paper.

Prior studies have found inconsistent results on the consolidation of general skill knowledge in the elderly population. Based on these studies, the length of the offline delay seems to influence the magnitude of consolidation in older adults (21, 45, 49). Concerning the prior studies, only Nemeth and Janacsek (45) employed a 24-h offline delay. They have found neither offline improvement nor forgetting in older adults, general skill knowledge did not change over the offline period. In contrast, we found offline improvement in 61- to 76-year olds following a 24-h delay and the magnitude of offline gains did not differ significantly between younger and older adults. However, it is worth noting that Bayesian evidence for offline gains in general skill knowledge was around 1 in the 61- to 76-year-old group, which means that we did not find evidence for either the null or the alternative hypothesis. Hence, further research is warranted to explore the consolidation of general skill knowledge in older adulthood.

A considerable amount of research has focused on the changes of different cognitive functions across the lifespan. The lifespan trajectory of several cognitive functions has been described as an inverted U-shape: these functions continuously mature through childhood and adolescence, then peak in young or middle adulthood and decline over the course of aging (e.g. 2, 3). Some functions peak in childhood and starts to decline as soon as adolescence or young adulthood (e.g. 9–11), whereas some functions remain intact in late adulthood as well (e.g. 12). The consolidation of statistical regularities seems to follow a different, age-invariant trajectory: the acquired statistical knowledge is comparably retained in all age groups from the age of 7 to 76 years. Consolidation of general skill knowledge also seems to follow an age-invariant trajectory: in this case, the offline improvement over the delay period is comparable across all age groups. Since the oldest adult in our study was 76 years old, future studies could provide further insights into how aging affects consolidation by involving individuals beyond this age as well.

One limitation of our study is the lack of interference design, which can be important for consolidation studies (80, 81). Consolidation can be defined both as stabilization of the acquired knowledge—usually evident as no change in performance or offline learning during the delay period—and as resistance to interference. A previous study with the ASRT task employed an interference design: Kóbor et al. (41) investigated the 1-year consolidation of statistical knowledge in healthy young adults. They showed successful retention as well as a resistance to interference. Hence, successful consolidation was expressed both by retention and resistance to interference in the same group of participants. Employing an interference design might also be relevant from a developmental perspective. Dorfberger et al. (82) showed similar consolidation in childhood and adulthood on the behavioral level using a motor learning task; however, a difference emerged concerning interference. Children seemed to be less susceptible to subsequent interference compared with adults, suggesting age-related differences in this aspect of consolidation. Therefore, future studies should employ an interference design to shed further light on the lifespan trajectory of memory consolidation.

Conducting large-scale studies involving participants from age groups across the lifespan is essential to characterize the development and aging of any cognitive functions. Inferences made from different sets of individual studies have a risk of different paradigms and designs confounding the conclusions. Here, we employed a lifespan approach in a cross-sectional design involving participants from 7 to 76 years using the same task across the whole sample. In conclusion, the present study demonstrated comparable consolidation of statistical and general skill knowledge following a 24-h offline delay across the lifespan, from 7- to 76-year olds. Our study offers evidence for age-invariance in these key cognitive functions.

## Supplementary Material

pgad037_Supplementary_Data

## Data Availability

Data used for the analyses reported in this paper are available on the following online repository: https://osf.io/cpwve/?view_only=0870aa34fa0443c5a2d34d8463a9dd6d.
