# Supplementary material for: Lifespan developmental invariance in memory consolidation: evidence from procedural memory
Source: PNAS Nexus. 2023 Feb 8;2(3):pgad037. doi: 10.1093/pnasnexus/pgad037 (PMC9991456; doi:10.1093/pnasnexus/pgad037)
Supplement: pgad037_Supplementary_Data [file pgad037_supplementary_data.docx]

**Supplementary Material** of the manuscript entitled

“Lifespan developmental invariance in memory consolidation: Evidence from procedural memory”

Eszter Tóth-Fáber, Dezso Nemeth, Karolina Janacsek

Table of contents

[Bayesian model comparisons for consolidation of statistical and general skill knowledge 2](#_Toc124335831)

[Supplementary Figures of offline change of statistical and general skill knowledge 3](#_Toc124335832)

[Results on learning 4](#_Toc124335833)

[Are there age-related differences in statistical learning in terms of RTs? 4](#_Toc124335834)

[Are there age-related differences in general skill learning in terms of RTs? 4](#_Toc124335835)

[Results on learning in terms of ratio scores 5](#_Toc124335836)

[Are there age-related differences in statistical learning in terms of ratio scores? 5](#_Toc124335837)

[Results on consolidation based on standardized reaction times 6](#_Toc124335838)

[Standardization with ratio scores 6](#_Toc124335839)

[Standardization with log-transformation 9](#_Toc124335840)

[Testing age-related differences in consolidation by estimating future performance in the Testing Phase by extrapolation 12](#_Toc124335841)

[Estimating statistical learning scores in the Testing Phase 12](#_Toc124335842)

[Estimating general skill learning scores in the Testing Phase 13](#_Toc124335843)

[Testing possible confounds influencing the consolidation of statistical and general skill knowledge 14](#_Toc124335844)

[Block-level analysis on the consolidation of statistical knowledge 14](#_Toc124335845)

[Block-level analysis on the consolidation of general skill knowledge 15](#_Toc124335846)

[The consolidation of general skill knowledge in terms of accuracy scores 16](#_Toc124335847)

# Bayesian model comparisons for consolidation of statistical and general skill knowledge

**Table S1.** Bayesian model comparisons for consolidation of statistical knowledge.

| **Models** | | **P(M)** | | **P(M\|data)** | | **BF_M_** | | **BF_01_** | | **error %** | |
| --- | --- | --- | --- | --- | --- | --- | --- | --- | --- | --- | --- |
| Null model (incl. subject) |  | 0.200 |  | 0.179 |  | 0.871 |  | 1.000 |  |  |  |
| Age group |  | 0.200 |  | 0.717 |  | 10.122 |  | 0.250 |  | 0.480 |  |
| Epoch + Age group |  | 0.200 |  | 0.083 |  | 0.363 |  | 2.150 |  | 1.807 |  |
| Epoch |  | 0.200 |  | 0.021 |  | 0.085 |  | 8.583 |  | 1.343 |  |
| Epoch + Age group + Epoch x Age group |  | 0.200 |  | 3.382e -4 |  | 0.001 |  | 528.860 |  | 1.477 |  |

*Notes.* All models include subject. The Models column denotes the predictors included in each model, the P(M) column the prior model probability, the P(M|data) column the posterior model probability, the BF_M_ column the posterior model odds, and the BF_01_ column the Bayes factors for each model compared to the null model. BF_01_ values between 1 and 3 indicate anecdotal evidence, values between 3 and 10 indicate substantial evidence and values larger than 10 indicate strong evidence for H_0_. Values between 1 and 1/3 suggest anecdotal evidence, values between 1/3 and 1/10 indicate substantial evidence, and values below 1/10 indicate strong evidence for H_1_. Values around 1 do not support either hypothesis. The error is an estimate of the numerical error in the computation of the Bayes factor.

**Table S2.** Bayesian model comparisons for consolidation of general skill knowledge.

| **Models** | | **P(M)** | | **P(M\|data)** | | **BF_M_** | | **BF_01_** | | **error %** | |
| --- | --- | --- | --- | --- | --- | --- | --- | --- | --- | --- | --- |
| Null model (incl. subject) |  | 0.200 |  | 2.556e -46 |  | 1.022e -45 |  | 1.000 |  |  |  |
| Epoch + Age group |  | 0.200 |  | 0.548 |  | 4.857 |  | 4.661e -46 |  | 1.032 |  |
| Epoch + Age group + Epoch x Age group |  | 0.200 |  | 0.452 |  | 3.295 |  | 5.659e -46 |  | 1.407 |  |
| Age group |  | 0.200 |  | 5.850e -18 |  | 2.340e -17 |  | 4.369e -29 |  | 0.328 |  |
| Epoch |  | 0.200 |  | 2.831e -29 |  | 1.132e -28 |  | 9.027e -18 |  | 0.964 |  |

*Notes.*  All models include subject. The Models column denotes the predictors included in each model, the P(M) column the prior model probability, the P(M|data) column the posterior model probability, the BF_M_ column the posterior model odds, and the BF_01_ column the Bayes factors for each model compared to the null model. BF_01_ values between 1 and 3 indicate anecdotal evidence, values between 3 and 10 indicate substantial evidence and values larger than 10 indicate strong evidence for H_0_. Values between 1 and 1/3 suggest anecdotal evidence, values between 1/3 and 1/10 indicate substantial evidence, and values below 1/10 indicate strong evidence for H_1_. Values around 1 do not support either hypothesis. The error is an estimate of the numerical error in the computation of the Bayes factor.

# Supplementary Figures of offline change of statistical and general skill knowledge


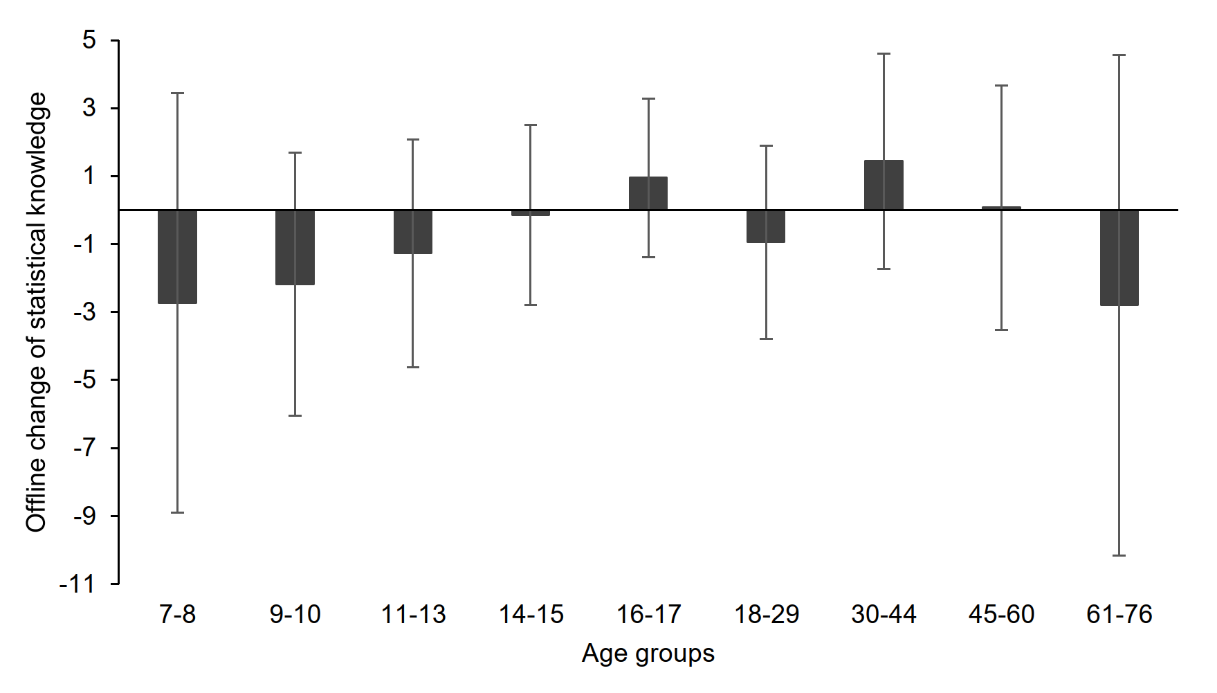


**Figure S1.** **Offline change of statistical knowledge over the 24-hour delay across the age groups.** Offline change scores were calculated by subtracting statistical knowledge values for the last epoch of the Learning Phase (Epoch 4) from those for the first epoch of the Testing Phase. Error bars denote the standard error of mean (SEM).


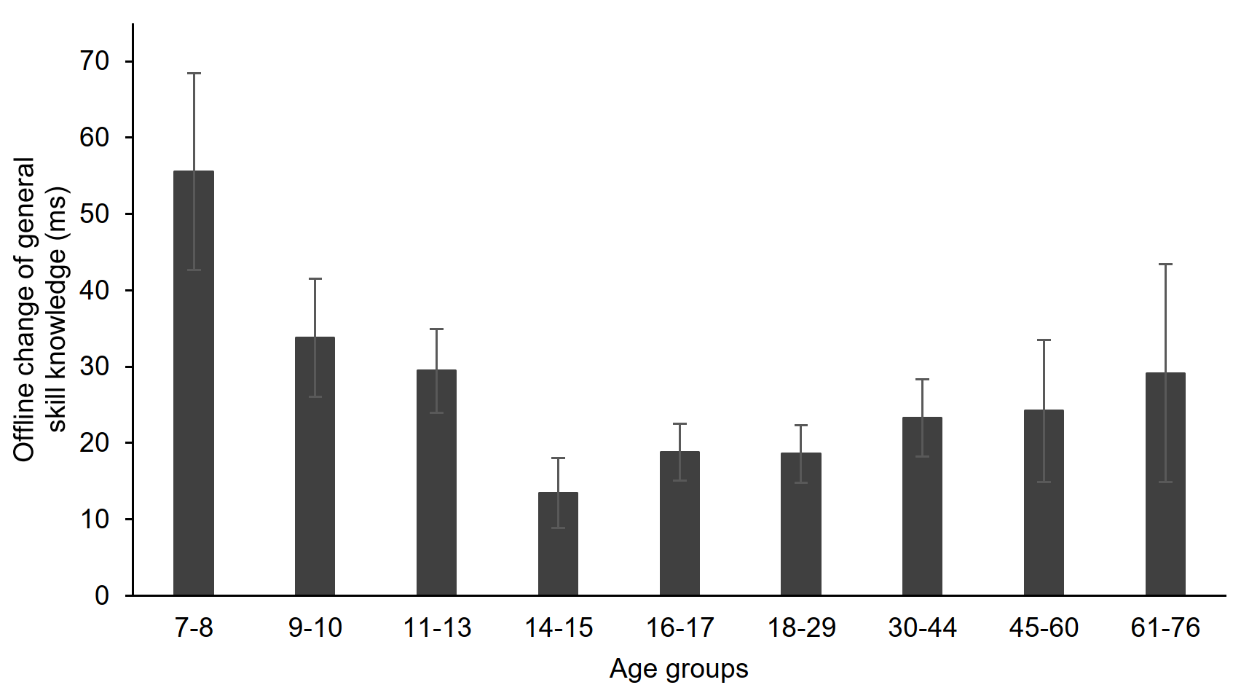


**Figure S2.** **Offline change of general skill knowledge over the 24-hour delay across the age groups.** Offline change scores were calculated by subtracting average RT values for the last epoch of the Learning Phase (Epoch 4) from those for the first epoch of the Testing Phase. Error bars denote the standard error of mean (SEM).

# Results on learning

## Are there age-related differences in statistical learning in terms of RTs?

To test whether the learning of statistical knowledge is age-variant, we submitted the statistical learning scores computed separately for the epochs of the Learning Phase (Epoch 1-4) to a mixed-design ANOVA with EPOCH (Epoch 1-4) as a within-subject factor and AGE GROUP as a between-subjects factor. The ANOVA revealed overall significant learning (main effect of INTERCEPT: *F*(1, 246) = 225.24, *p* < .001, *η*_p_^2^ = 0.48) and an overall increase of statistical learning score across the epochs (main effect of EPOCH: *F*(3, 738) = 7.63, *p* < .001, *η*_p_^2^ = 0.03). The trajectory of the increase was similar across the age groups (AGE GROUP x EPOCH interaction: *F*(24, 738) = 0.88, *p* = 0.63, *η*_p_^2^ = 0.03). Importantly, the ANOVA showed significant differences in overall learning across the age groups (main effect of AGE GROUP: *F*(8, 246) = 2.69, *p* = .008, *η*_p_^2^ = 0.08). Learning gradually decreased with age. In detail, the 7-8-year-olds showed better learning than participants over 14 (*p* < .042), the 9-10-year-olds showed better learning than participants over 16 (*p* < .04) and the 11-13-year-olds showed better learning than participants over 18 (*p* < .045). Over the age of 14, learning scores are comparable across the age groups (*p* > .32).

## Are there age-related differences in general skill learning in terms of RTs?

To test whether general skill learning is age-variant, we submitted the median RTs of the epochs in the Learning Phase (Epoch 1-4) to a mixed-design ANOVA with EPOCH (Epoch 1-4) as a within-subject factor and AGE GROUP as a between-subject factor. The ANOVA showed that over groups, median RTs decreased as the task progressed (main effect of EPOCH: *F*(3, 738) = 206.43, *p* < .001, *η*_p_^2^ = 0.46) and median RTs differed significantly across age groups (main effect of AGE GROUP: *F*(8, 246) = 27.90, *p* < .001, *η*_p_^2^ = 0.48), revealing a U-shaped trajectory with highest RTs in the youngest and oldest age groups. Moreover, the trajectory of general skill learning differed across the age groups (AGE GROUP x EPOCH interaction: *F*(24, 738) = 5.68, *p* < .001, *η*_p_^2^ = 0.16). We calculated the amount of change in RTs from Epoch 1 to Epoch 4 by subtracting median RTs in Epoch 4 from median RTs in Epoch 1. This way, higher scores indicate a steeper decrease of reaction times, that is, better general skill learning. A follow-up one-way ANOVA on this score showed that 7-8-year-olds exhibited the greatest general skill learning, which significantly differed from the other age groups’ (*p*s < .01), except for the 61-76-year-old group (*p* = .34). The 9-10-year-olds showed smaller speed-up than the 7-8-year-olds, a comparable speed-up to the 11-13-year-olds and a higher speed-up than the other age groups between 14 and 60 years of age (*p* < .039). From 14 to 60 years of age, general skill learning was comparable across the age groups (*p* > .41). The 61-76-year-olds’ general skill learning was significantly smaller than the other age groups (*p* < .006), expect for the 7-8-year-olds (*p* = .34) and 9-10-year-olds (*p* = .108).

# Results on learning in terms of ratio scores

Due to the baseline RT differences across the age groups, we conducted additional ANOVA on ratio scores to test statistical learning while controlling for RT differences across the groups (for details on the standardization process, see Statistical analysis section of the manuscript). As the effect of standardization on general skill learning was comprehensively tested on this database in the study of [Juhasz, et al. ^1^](#_ENREF_1), here, we only report results on statistical learning in terms of ratio scores.

## Are there age-related differences in statistical learning in terms of ratio scores?

Identically to the raw learning scores, we submitted the ratio scores to a mixed-design ANOVA with EPOCH (Epoch 1-4) as a within-subject factor and AGE GROUP as a between-subjects factor. The ANOVA revealed overall significant learning (main effect of INTERCEPT: *F*(1, 246) = 278.07, *p* < .001, *η*_p_^2^ = 0.53) and an overall increase of statistical learning score across the epochs (main effect of EPOCH: *F*(3, 738) = 12.22, *p* < .001, *η*_p_^2^ = 0.05). The trajectory of the increase was similar across the age groups (AGE GROUP x EPOCH interaction: *F*(24, 738) = 0.93, *p* = 0.56, *η*_p_^2^ = 0.03). The ANOVA also revealed significant differences in overall learning across the age groups (main effect of AGE GROUP: *F*(8, 246) = 2.50, *p* = .01, *η*_p_^2^ = 0.08), however, the differences were not identical to those of the analysis on raw statistical learning scores. Standardized statistical learning scores were comparable between the age of 7 and 29 (*p* > .096, BF_01_ > 1.859). The 30-44-year-old group exhibited decreased learning compared to 9-10-year-olds (*p* = .05, BF_01_ = 1.142) and 11-13-year-olds (*p* = .02, BF_01_ = 0.485), but there was no significant difference in the learning scores between the 30-44-year-old group and the age groups between 14 and 29 years (*p*s > .087, BF_01_ > 1.438). The 45-60-year-old and 61-76-year-old group showed decreased learning than almost all the age groups under the age of 15 (*p* < .052, BF_01_ < 0.828, except for the 7-8-year-olds vs. 61-76-year-olds, where *p* = .057, BF_01_ = 0.654).

# Results on consolidation based on standardized reaction times

It is well-established that children and older adults respond with slower reaction times (RTs) overall[^1^](#_ENREF_1)^,^[^2^](#_ENREF_2). Hence, we conducted additional ANOVAs on standardized RTs to probe the retention of statistical and general skill knowledge while controlling for average RT differences across age groups. We standardized the data in two different ways: we calculated (1) ratio scores and (2) log-transformed RT data (for details on the standardization process, see Statistical analysis section of the manuscript). Here, we report the exact statistics of the analyses on standardized RTs.

## Standardization with ratio scores

**Do age groups differ in consolidation of statistical knowledge in terms of ratio scores?**

To rule out the possibility of average RT differences among the age groups confounding our results, we tested the consolidation of statistical knowledge on ratio scores as well. We contrasted statistical learning scores computed from ratio scores for the last epoch of the Learning Phase (Epoch 4) with the learning scores computed for the first of epoch of the Testing Phase (Epoch 5) and submitted these scores to a mixed-design ANOVA with EPOCH (Epoch 4 vs Epoch 5) as a within-subject factor and AGE GROUP as a between-subjects factor. The ANOVA showed overall significant statistical knowledge (main effect of INTERCEPT: *F*(1, 246) = 388.30, *p* < 0.001, *η*_p_^2^ = 0.61) and significant differences in overall learning across age groups (main effect of AGE GROUP: *F*(8, 246) = 4.70, *p* < 0.001, *η*_p_^2^ = 0.13). Importantly, statistical knowledge appears to be retained over the 24-hour delay period with no significant change between the end of the Learning Phase and the Testing Phase (main effect of EPOCH: *F*(1, 246) = 0.25, *p* = 0.62, *η*_p_^2^ = 0.001). Moreover, no age group differences emerged in the retention of the statistical knowledge (non-significant EPOCH x AGE GROUP interaction: *F*(8, 246) = 0.18, *p* = 0.99, *η*_p_^2^ = 0.006): all age groups retained the acquired knowledge over the 24-hour delay period (all *p*s > .37). Bayesian mixed-design ANOVA on the standardized scores also supported the finding of the frequentist ANOVA by showing that the main effect of EPOCH and EPOCH x AGE GROUP interaction should be excluded from the model (Table S3 and S4). Thus, this analysis also suggests the successful retention of statistical knowledge over the 24-hour delay period in all age groups.

**Table S3.** Analysis of effects of Bayesian ANOVA for consolidation of standardized statistical knowledge in terms of ratio scores.

| **Effects** | **P(incl)** | **P(incl\|data)** | **BF_exclusion_** |
| --- | --- | --- | --- |
| Epoch | 0.400 | 0.099 | 9.081 |
| Age group | 0.400 | 0.998 | 0.002 |
| Epoch x Age group | 0.200 | 4.196e-4 | 235.891 |

Notes. The Effects column denotes the main effects and interaction. The P(incl) column indicates the prior inclusion probability and the P(incl|data) denotes the posterior inclusion probability. The BF_exclusion_ column shows the exclusion Bayes Factors. BF_exclusion_ values below 1 support the inclusion and values above 1 the exclusion of the given factor.

**Table S4.** Bayesian model comparisons for standardized statistical knowledge in terms of ratio scores.

| **Models** | | **P(M)** | | **P(M\|data)** | | **BF_M_** | | **BF_01_** | | **error %** | |
| --- | --- | --- | --- | --- | --- | --- | --- | --- | --- | --- | --- |
| Null model (incl. subject) |  | 0.200 |  | 0.002 |  | 0.006 |  | 1.000 |  |  |  |
| Age group |  | 0.200 |  | 0.899 |  | 35.556 |  | 0.002 |  | 0.494 |  |
| Epoch + Age group |  | 0.200 |  | 0.099 |  | 0.439 |  | 0.016 |  | 1.699 |  |
| Epoch + Age group + Epoch x Age group |  | 0.200 |  | 4.196e -4 |  | 0.002 |  | 3.690 |  | 0.965 |  |
| Epoch |  | 0.200 |  | 1.686e -4 |  | 6.747e -4 |  | 9.182 |  | 1.760 |  |

*Notes.*  All models include subject. The Models column denotes the predictors included in each model, the P(M) column the prior model probability, the P(M|data) column the posterior model probability, the BF_M_ column the posterior model odds, and the BF_01_ column the Bayes factors for each model compared to the null model. BF_01_ values between 1 and 3 indicate anecdotal evidence, values between 3 and 10 indicate substantial evidence and values larger than 10 indicate strong evidence for H_0_. Values between 1 and 1/3 suggest anecdotal evidence, values between 1/3 and 1/10 indicate substantial evidence, and values below 1/10 indicate strong evidence for H_1_. Values around 1 do not support either hypothesis. The error is an estimate of the numerical error in the computation of the Bayes factor.

**Do age groups differ in consolidation of general skill knowledge in terms of ratio scores?**

Similarly to statistical knowledge, we tested consolidation of general skill knowledge (defined as overall RT changes) over the 24-hour delay period using ratio scores as well. We ran an ANOVA on ratio scores with EPOCH (Epoch 4 vs Epoch 5) as a within-subject factor and AGE GROUP as a between-subjects factor. The ANOVA revealed that RTs significantly decreased over the 24-hour delay (main effect of EPOCH: *F*(1, 246) = 149.35, *p* < 0.001, *η*_p_^2^ = 0.38), thus participants responded faster in the Testing Phase compared to the end of the Learning Phase (significant speed-up in all age groups: all *p*s < 0.007). The amount of RT speed-up over the delay period was similar across age groups (non-significant EPOCH x AGE GROUP interaction: *F*(8, 246) = 1.23, *p* = 0.28, *η*_p_^2^ = 0.04; Supplementary Fig. 3). Bayesian mixed-design ANOVA showed evidence for the inclusion of the main effect of EPOCH and the exclusion of the EPOCH x AGE GROUP interaction (Table S5 and S6), suggesting an overall speed-up over the delay period and a lack of differences in the amount of speed-up across age groups.


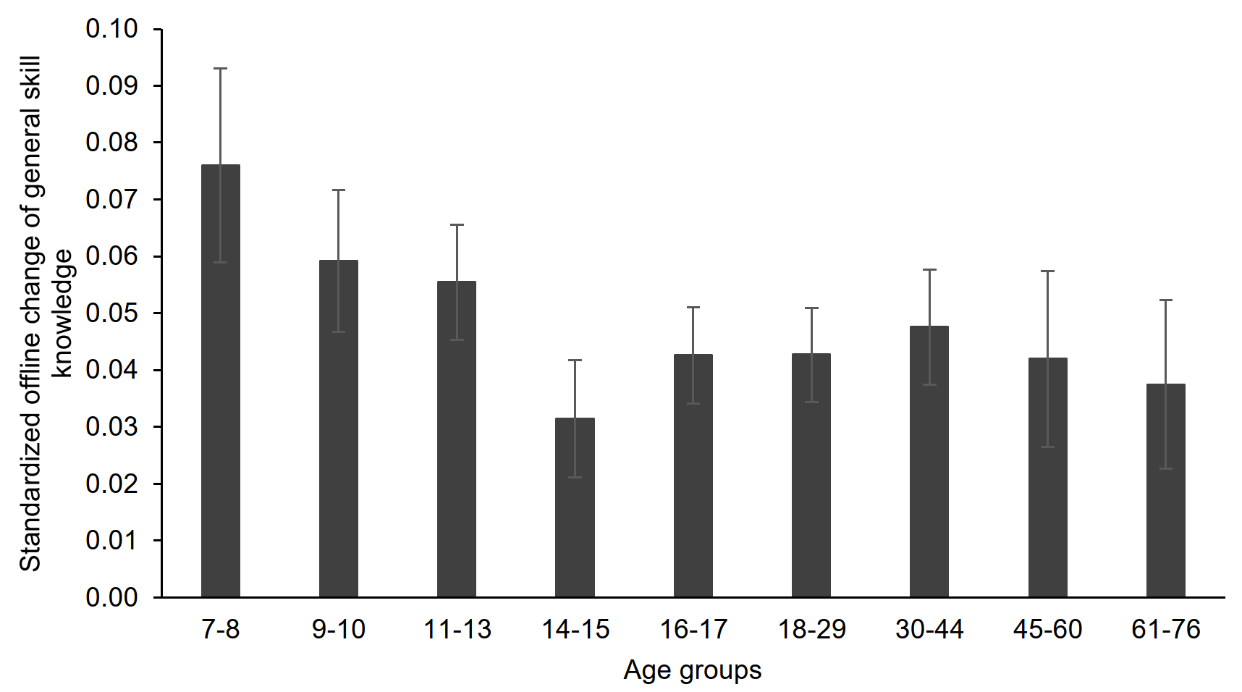


**Figure S3.** **Standardized offline change of general skill knowledge over the 24-hour delay across the age groups.** Standardized offline change scores were calculated by subtracting standardized average RT values for the last epoch of the Learning Phase (Epoch 4) from those for the first epoch of the Testing Phase. Error bars denote the SEM.

**Table S5.** Analysis of effects of Bayesian ANOVA for consolidation of standardized general skill knowledge in terms of ratio scores.

| **Effects** | **P(incl)** | **P(incl\|data)** | **BF_exclusion_** |
| --- | --- | --- | --- |
| Epoch | 0.400 | 0.945 | 1.728e-24 |
| Age group | 0.400 | 0.945 | 1.391e-6 |
| Epoch x Age group | 0.200 | 0.055 | 17.306 |

Notes. The Effects column denotes the main effects and interaction. The P(incl) column indicates the prior inclusion probability and the P(incl|data) denotes the posterior inclusion probability. The BF_exclusion_ column shows the exclusion Bayes Factors. BF_exclusion_ values below 1 support the inclusion and values above 1 the exclusion of the given factor.

**Table S6.** Bayesian model comparisons for standardized general skill knowledge in terms of ratio scores.

| **Models** | | **P(M)** | | **P(M\|data)** | | **BF_M_** | | **BF_01_** | | **error %** | |
| --- | --- | --- | --- | --- | --- | --- | --- | --- | --- | --- | --- |
| Null model (incl. subject) |  | 0.200 |  | 2.010e -30 |  | 8.038e -30 |  | 1.000 |  |  |  |
| Epoch + Age group |  | 0.200 |  | 0.945 |  | 69.222 |  | 2.126e -30 |  | 1.182 |  |
| Epoch + Age group + Epoch x Age group |  | 0.200 |  | 0.055 |  | 0.231 |  | 3.679e -29 |  | 2.024 |  |
| Epoch |  | 0.200 |  | 1.315e -6 |  | 5.262e -6 |  | 1.528e -24 |  | 2.009 |  |
| Age group |  | 0.200 |  | 1.634e -24 |  | 6.536e -24 |  | 1.230e -6 |  | 0.861 |  |

*Notes.* All models include subject. The Models column denotes the predictors included in each model, the P(M) column the prior model probability, the P(M|data) column the posterior model probability, the BF_M_ column the posterior model odds, and the BF_01_ column the Bayes factors for each model compared to the null model. BF_01_ values between 1 and 3 indicate anecdotal evidence, values between 3 and 10 indicate substantial evidence and values larger than 10 indicate strong evidence for H_0_. Values between 1 and 1/3 suggest anecdotal evidence, values between 1/3 and 1/10 indicate substantial evidence, and values below 1/10 indicate strong evidence for H_1_. Values around 1 do not support either hypothesis. The error is an estimate of the numerical error in the computation of the Bayes factor.

## Standardization with log-transformation

For log-transformed RT data, we conducted identical ANOVAs as the ones presented in the manuscript and the ones for ratio scores presented in the supplementary materials, separately for statistical knowledge and general skill knowledge scores. Importantly, log-transformed standardization showed identical results as ratio score standardization both for statistical knowledge scores and general skill knowledge scores, both in frequentist and Bayesian ANOVAs. The results on statistical knowledge are presented in Table S7, S8 and S9; and the results on general skill knowledge are presented in Table S10, S11 and S12.

**Table S7.** The results of mixed-design ANOVA on the log-transformed statistical knowledge.

| Effects | F | p | *η*_p_^2^ |
| --- | --- | --- | --- |
| INTERCEPT | 380.35 | < 0.001 | 0.61 |
| AGE GROUP | 4.44 | < 0.001 | 0.13 |
| EPOCH | 1.40 | 0.24 | 0.006 |
| EPOCH x AGE GROUP | 0.80 | 0.60 | 0.025 |

*Notes.* Statistical knowledge is retained over the delay period in all age groups, with no age group differences in retention.

**Table S8.** Analysis of effects of Bayesian ANOVA for consolidation of log-transformed statistical knowledge.

| **Effects** | **P(incl)** | **P(incl\|data)** | **BF_exclusion_** |
| --- | --- | --- | --- |
| Epoch | 0.400 | 0.158 | 5.307 |
| Age group | 0.400 | 0.992 | 0.005 |
| Epoch x Age group | 0.200 | 0.003 | 46.144 |

*Notes.* The Effects column denotes the main effects and interaction. The P(incl) column indicates the prior inclusion probability and the P(incl|data) denotes the posterior inclusion probability. The BF_exclusion_ column shows the exclusion Bayes Factors. BF_exclusion_ values below 1 support the inclusion and values above 1 the exclusion of the given factor.

**Table S9.** Bayesian model comparisons for log-transformed statistical knowledge.

| **Models** | | **P(M)** | | **P(M\|data)** | | **BF_M_** | | **BF_01_** | | **error %** | |
| --- | --- | --- | --- | --- | --- | --- | --- | --- | --- | --- | --- |
| Null model (incl. subject) |  | 0.200 |  | 0.004 |  | 0.015 |  | 1.000 |  |  |  |
| Age group |  | 0.200 |  | 0.835 |  | 20.211 |  | 0.005 |  | 0.850 |  |
| Epoch + Age group |  | 0.200 |  | 0.157 |  | 0.747 |  | 0.024 |  | 1.953 |  |
| Epoch + Age group + Epoch x Age group |  | 0.200 |  | 0.003 |  | 0.014 |  | 1.110 |  | 1.550 |  |
| Epoch |  | 0.200 |  | 7.079e-4 |  | 0.003 |  | 5.345 |  | 1.865 |  |

*Notes.* All models include subject. The Models column denotes the predictors included in each model, the P(M) column the prior model probability, the P(M|data) column the posterior model probability, the BF_M_ column the posterior model odds, and the BF_01_ column the Bayes factors for each model compared to the null model. BF_01_ values between 1 and 3 indicate anecdotal evidence, values between 3 and 10 indicate substantial evidence and values larger than 10 indicate strong evidence for H_0_. Values between 1 and 1/3 suggest anecdotal evidence, values between 1/3 and 1/10 indicate substantial evidence, and values below 1/10 indicate strong evidence for H_1_. Values around 1 do not support either hypothesis. The error is an estimate of the numerical error in the computation of the Bayes factor.

**Table S10.** The results of mixed-design ANOVA on the log-transformed general skill knowledge.

| Effects | F | p | *η*_p_^2^ |
| --- | --- | --- | --- |
| AGE GROUP | 31.53 | < 0.001 | 0.51 |
| EPOCH | 143.10 | < 0.001 | 0.37 |
| EPOCH x AGE GROUP | 1.33 | 0.23 | 0.04 |

*Notes.* All age groups showed offline learning (all *p*s < .050) and the speed-up over the delay period was similar across the age groups.

**Table S11.** Analysis of effects of Bayesian ANOVA for consolidation of log-transformed general skill knowledge.

| **Effects** | **P(incl)** | **P(incl\|data)** | **BF_exclusion_** |
| --- | --- | --- | --- |
| Epoch | 0.400 | 0.932 | 9.798e-24 |
| Age group | 0.400 | 0.932 | 6.645e-31 |
| Epoch x Age group | 0.200 | 0.068 | 13.638 |

*Notes.* The Effects column denotes the main effects and interaction. The P(incl) column indicates the prior inclusion probability and the P(incl|data) denotes the posterior inclusion probability. The BF_exclusion_ column shows the exclusion Bayes Factors. BF_exclusion_ values below 1 support the inclusion and values above 1 the exclusion of the given factor.

**Table S12.** Bayesian model comparisons for log-transformed general skill knowledge.

| **Models** | | **P(M)** | | **P(M\|data)** | | **BF_M_** | | **BF_01_** | | **error %** | |
| --- | --- | --- | --- | --- | --- | --- | --- | --- | --- | --- | --- |
| Null model (incl. subject) |  | 0.200 |  | 4.944e-54 |  | 1.977e-53 |  | 1.000 |  |  |  |
| Epoch + Age group |  | 0.200 |  | 0.932 |  | 54.552 |  | 5.306e-54 |  | 1.203 |  |
| Epoch + Age group + Epoch x Age group |  | 0.200 |  | 0.068 |  | 0.293 |  | 7.237e-53 |  | 1.700 |  |
| Age group |  | 0.200 |  | 9.129e-24 |  | 3.652e-23 |  | 5.415e-31 |  | 0.430 |  |
| Epoch |  | 0.200 |  | 6.191e-31 |  | 2.477e-30 |  | 7.985e-24 |  | 0.937 |  |

*Notes.* All models include subject. The Models column denotes the predictors included in each model, the P(M) column the prior model probability, the P(M|data) column the posterior model probability, the BF_M_ column the posterior model odds, and the BF_01_ column the Bayes factors for each model compared to the null model. BF_01_ values between 1 and 3 indicate anecdotal evidence, values between 3 and 10 indicate substantial evidence and values larger than 10 indicate strong evidence for H_0_. Values between 1 and 1/3 suggest anecdotal evidence, values between 1/3 and 1/10 indicate substantial evidence, and values below 1/10 indicate strong evidence for H_1_. Values around 1 do not support either hypothesis. The error is an estimate of the numerical error in the computation of the Bayes factor.

# Testing age-related differences in consolidation by estimating future performance in the Testing Phase by extrapolation

## Estimating statistical learning scores in the Testing Phase

**Table S13.** The results of the mixed-design ANOVA on the difference between the predicted and observed statistical learning scores.

| Effects | F | p | *η*_p_^2^ |
| --- | --- | --- | --- |
| INTERCEPT | 16.15 | < 0.001 | 0.06 |
| BLOCK | 1.56 | 0.18 | 0.006 |
| AGE GROUP | 1.53 | 0.15 | 0.05 |
| BLOCK x AGE GROUP | 0.70 | 0.90 | 0.02 |

**Table S14.** Analysis of effects of the Bayesian ANOVA on the difference between the predicted and observed statistical learning scores.

| **Effects** | **P(incl)** | **P(incl\|data)** | **BF_exclusion_** |
| --- | --- | --- | --- |
| Block | 0.400 | 0.013 | 73.908 |
| Age group | 0.400 | 0.005 | 206.070 |
| Block x Age group | 0.200 | 1.270e-8 | 5234.761 |

*Notes.* The Effects column denotes the main effects and interaction. The P(incl) column indicates the prior inclusion probability and the P(incl|data) denotes the posterior inclusion probability. The BF_exclusion_ column shows the exclusion Bayes Factors. BF_exclusion_ values below 1 support the inclusion and values above 1 the exclusion of the given factor.

**Table S15.** Bayesian model comparisons for the difference between the predicted and observed statistical learning scores.

| **Models** | | **P(M)** | | **P(M\|data)** | | **BF_M_** | | **BF_01_** | | **error %** | |
| --- | --- | --- | --- | --- | --- | --- | --- | --- | --- | --- | --- |
| Null model (incl. subject) |  | 0.200 |  |  | 0.982 | 216.843 |  | 1.000 |  |  |  |
| Block |  | 0.200 |  |  | 0.013 | 0.054 |  | 73.920 |  | 2.037 |  |
| Age group |  | 0.200 |  |  | 0.005 | 0.019 |  | 206.157 |  | 0.280 |  |
| Block + Age group |  | 0.200 |  |  | 6.647e-5 | 2.659e-4 |  | 14771.791 |  | 1.761 |  |
| Block + Age group + Block x Age group |  | 0.200 |  |  | 1.270e-8 | 5.079e-8 |  | 7.733e+7 |  | 0.687 |  |

*Notes.* All models include subject. The Models column denotes the predictors included in each model, the P(M) column the prior model probability, the P(M|data) column the posterior model probability, the BF_M_ column the posterior model odds, and the BF_01_ column the Bayes factors for each model compared to the null model. BF_01_ values between 1 and 3 indicate anecdotal evidence, values between 3 and 10 indicate substantial evidence and values larger than 10 indicate strong evidence for H_0_. Values between 1 and 1/3 suggest anecdotal evidence, values between 1/3 and 1/10 indicate substantial evidence, and values below 1/10 indicate strong evidence for H_1_. Values around 1 do not support either hypothesis. The error is an estimate of the numerical error in the computation of the Bayes factor.

## Estimating general skill learning scores in the Testing Phase

**Table S16.** The results of the mixed-design ANOVA on the difference between the predicted and observed general skill learning scores.

| Effects | F | p | *η*_p_^2^ |
| --- | --- | --- | --- |
| INTERCEPT | 14.59 | < 0.001 | 0.06 |
| BLOCK | 6.84 | < 0.001 | 0.03 |
| AGE GROUP | 0.47 | 0.88 | 0.02 |
| BLOCK x AGE GROUP | 1.00 | 0.46 | 0.03 |

**Table S17.** Analysis of effects of the Bayesian ANOVA on the difference between the predicted and observed general skill learning scores.

| **Effects** | **P(incl)** | **P(incl\|data)** | **BF_exclusion_** |
| --- | --- | --- | --- |
| Block | 0.400 | 0.987 | 0.013 |
| Age group | 0.400 | 0.186 | 4.376 |
| Block x Age group | 0.200 | 6.190e-4 | 296.538 |

*Notes.* The Effects column denotes the main effects and interaction. The P(incl) column indicates the prior inclusion probability and the P(incl|data) denotes the posterior inclusion probability. The BF_exclusion_ column shows the exclusion Bayes Factors. BF_exclusion_ values below 1 support the inclusion and values above 1 the exclusion of the given factor.

**Table S18.** Bayesian model comparisons for the difference between the predicted and observed general skill learning scores.

| **Models** | | **P(M)** | | **P(M\|data)** | | **BF_M_** | | **BF_01_** | | **error %** | |
| --- | --- | --- | --- | --- | --- | --- | --- | --- | --- | --- | --- |
| Null model (incl. subject) |  | 0.200 |  | 0.010 |  | 0.041 |  | 1.000 |  |  |  |
| Block |  | 0.200 |  | 0.803 |  | 16.347 |  | 0.013 |  | 0.975 |  |
| Block + Age group |  | 0.200 |  | 0.184 |  | 0.899 |  | 0.055 |  | 14.114 |  |
| Age group |  | 0.200 |  | 0.002 |  | 0.009 |  | 4.332 |  | 7.148 |  |
| Block + Age group + Block x Age group |  | 0.200 |  | 6.190e-4 |  | 0.002 |  | 16.272 |  | 66.534 |  |

*Notes.* All models include subject. The Models column denotes the predictors included in each model, the P(M) column the prior model probability, the P(M|data) column the posterior model probability, the BF_M_ column the posterior model odds, and the BF_01_ column the Bayes factors for each model compared to the null model. BF_01_ values between 1 and 3 indicate anecdotal evidence, values between 3 and 10 indicate substantial evidence and values larger than 10 indicate strong evidence for H_0_. Values between 1 and 1/3 suggest anecdotal evidence, values between 1/3 and 1/10 indicate substantial evidence, and values below 1/10 indicate strong evidence for H_1_. Values around 1 do not support either hypothesis. The error is an estimate of the numerical error in the computation of the Bayes factor.

# Testing possible confounds influencing the consolidation of statistical and general skill knowledge

## Block-level analysis on the consolidation of statistical knowledge

To test the possible confounding effect of averaging over the last five blocks of the Learning Phase and the first five blocks of the Testing Phase[^3^](#_ENREF_3), we contrasted performance in the last block of the Learning Phase (Block 20) and the first block is the Testing Phase (Block 21). We ran a mixed-design ANOVA on statistical learning scores with BLOCK (Block 20 vs Block 21) as a within-subject factor and AGE GROUP as a between-subjects factor. The analysis revealed that, on the group level, some degree of forgetting cannot be ruled out as the main effect of BLOCK was at the trend-level (*F*(1, 246) = 3.54, *p* = 0.06, *η*_p_^2^ = 0.01), learning scores were lower in Block 21 (M = 9.28 ms) than in Block 20 (M = 15.38 ms). Importantly, no significant age-related differences were detected in the retention of statistical knowledge (non-significant BLOCK x AGE GROUP interaction: *F*(8, 246) = 1.67, *p* = 0.11, *η*_p_^2^ = 0.05).

We also ran a Bayesian mixed-design ANOVA identical to the frequentist one. The ANOVA suggested that regarding the main effect of BLOCK, the data is not conclusive, the Bayes Factor was around 1, not supporting either the null or the alternative hypothesis (Table S19 and S20). Moreover, the lack of age-related differences in retention was supported by the Bayesian ANOVA (Table S19 and S20), corroborating the results of the frequentist ANOVA.

As noted in the main text, the analysis of block-wise data in the ASRT task should be interpreted carefully due to the relatively low number of trials. Statistical learning scores are calculated as difference scores between the high- and low-probability trials after excluding the first five random practice trials at the beginning of the block, erroneous responses as well as trills and repetitions from the 85 trials that are presented in a block. Hence, aggregated (mostly epoch-level) data has been used to characterize learning in the ASRT task since its inception because it enables to track the trajectory of learning while simultaneously decreasing the effect of noise in the learning scores to an acceptable level[^4-6^](#_ENREF_4).

**Table S19.** Analysis of effects of the Bayesian ANOVA on statistical knowledge in the last block of the Learning Phase and in the first block of the Testing Phase.

| **Effects** | **P(incl)** | **P(incl\|data)** | **BF_exclusion_** |
| --- | --- | --- | --- |
| Block | 0.400 | 0.419 | 1.380 |
| Age group | 0.400 | 0.020 | 50.135 |
| Block x Age group | 0.200 | 0.002 | 3.762 |

*Notes.* The Effects column denotes the main effects and interaction. The P(incl) column indicates the prior inclusion probability and the P(incl|data) denotes the posterior inclusion probability. The BF_exclusion_ column shows the exclusion Bayes Factors. BF_exclusion_ values below 1 support the inclusion and values above 1 the exclusion of the given factor.

**Table S20.** Bayesian model comparisons for statistical knowledge in the last block of the Learning Phase and in the first block of the Testing Phase.

| **Models** | | **P(M)** | | **P(M\|data)** | | **BF_M_** | | **BF_01_** | | **error %** | |
| --- | --- | --- | --- | --- | --- | --- | --- | --- | --- | --- | --- |
| Null model (incl. subject) |  | 0.200 |  | 0.567 |  | 5.245 |  | 1.000 |  |  |  |
| Block |  | 0.200 |  | 0.411 |  | 2.790 |  | 1.381 |  | 0.996 |  |
| Age group |  | 0.200 |  | 0.011 |  | 0.045 |  | 50.798 |  | 0.269 |  |
| Block + Age group |  | 0.200 |  | 0.008 |  | 0.034 |  | 67.997 |  | 1.591 |  |
| Block + Age group + Block x Age group |  | 0.200 |  | 0.002 |  | 0.009 |  | 255.803 |  | 1.778 |  |

*Notes.* All models include subject. The Models column denotes the predictors included in each model, the P(M) column the prior model probability, the P(M|data) column the posterior model probability, the BF_M_ column the posterior model odds, and the BF_01_ column the Bayes factors for each model compared to the null model. BF_01_ values between 1 and 3 indicate anecdotal evidence, values between 3 and 10 indicate substantial evidence and values larger than 10 indicate strong evidence for H_0_. Values between 1 and 1/3 suggest anecdotal evidence, values between 1/3 and 1/10 indicate substantial evidence, and values below 1/10 indicate strong evidence for H_1_. Values around 1 do not support either hypothesis. The error is an estimate of the numerical error in the computation of the Bayes factor.

## Block-level analysis on the consolidation of general skill knowledge

To test whether practice-dependent changes in the Testing Phase influenced the offline learning of general skill knowledge over the 24-hour offline delay, we compared performance in the last block of the Learning Phase (Block 20) and the first block of the Testing Phase (Block 21). In detail, we contrasted general skill knowledge over the delay period with a mixed-design ANOVA on median RTs (i.e., RTs irrespective of the probabilities of events) with BLOCK (Block 20 vs Block 21) as a within-subject factor and AGE GROUP as a between-subject factor. The analysis showed that on the group level, median RTs significantly decreased over the 24-hour delay (main effect of BLOCK: *F*(1, 246) = 11.00, *p* = 0.001, *η*_p_^2^ = 0.04), participants responded faster in the first block of the Testing Phase compared to the last block of the Learning Phase. The amount of speed-up was comparable across the age groups (as suggested by the non-significant BLOCK x AGE GROUP interaction: *F*(8, 246) = 1.05, *p* = 0.40, *η*_p_^2^ = 0.03). This suggests that practice-dependent changes did not influence offline learning of general skill knowledge over the offline delay.

To further investigate this effect, we conducted an additional ANOVA with PHASE (Learning Phase vs. Testing Phase) and BLOCK (1-5) as within-subject factors and AGE GROUP as a between-subjects factor. This way, we could compare how performance changed across Block 1-5 (first five blocks of the Learning Phase) and Block 21-25 (first five blocks of the Testing Phase). A similar increase in RTs in Block 1-5 and Block 21-25 would suggest that the offline learning showed over the offline delay could be explained by the additional practice in the Testing Phase rather than consolidation. The results are shown in Table S21.

**Table S21.** The results of the mixed-design ANOVA on general skill knowledge in the first five blocks of the Learning Phase and in the first five blocks of the Testing Phase.

| Effects | F | p | *η*_p_^2^ |
| --- | --- | --- | --- |
| AGE GROUP | 28.51 | < 0.001 | 0.48 |
| PHASE | 554.79 | < 0.001 | 0.69 |
| BLOCK | 82.31 | < 0.001 | 0.25 |
| AGE GROUP x PHASE | 12.60 | < 0.001 | 0.69 |
| AGE GROUP x BLOCK | 2.48 | < 0.001 | 0.08 |
| PHASE x BLOCK | 58.06 | < 0.001 | 0.19 |
| AGE GROUP x PHASE x BLOCK | 2.26 | 0.001 | 0.07 |

The significant PHASE x BLOCK interaction suggests that the change in RTs across the blocks differed in the Learning and Testing Phases. Post-hoc analyses suggest that in the Learning Phase, there was a steeper increase in RTs (*M*_Block 1_ = 625.56 ms; *M*_Block 5_ = 544.44 ms, difference: 81.12 ms) than in the Testing Phase (*M*_Block 21_ = 466.94 ms, *M*_Block 25_ = 454.49 ms, difference: 12.45 ms). In the Learning Phase, all blocks differed from each other (all *p*s < 0.003) expect for Block 4 vs. 5 (*p* = 0.27). In the Testing Phase, RTs were slower in Block 21 compared to the remaining blocks (all *p*s < 0.001), but the remaining blocks did not differ from each other (all *p*s > 0.34). The steeper increase in RTs at the beginning of the Learning Phase than at the beginning of the Testing Phase suggest that the observed offline learning of general skill knowledge over the 24-hour offline delay was not due to further practice-dependent changes in the Testing Phase.

The significant AGE GROUP x PHASE x BLOCK interaction suggests that the trajectory of performance change across the blocks in the Learning and Testing Phases differed in the age groups. To disentangle this effect, we ran two follow-up ANOVAs with BLOCK as a within-subject factor and AGE GROUP as a between-subject factor separately for the two phases. In the Learning Phase, the AGE GROUP x BLOCK interaction was significant: there was a steeper increase in RTs in the 7-8, 45-60 and 61-76-year-old groups compared to the other groups. In the Testing Phase, however, AGE GROUP x BLOCK interaction did not reach significance, suggesting that the trajectory of performance change was similar across the age groups.

## The consolidation of general skill knowledge in terms of accuracy scores

To test whether offline learning in terms of reaction times could be influenced by decreased accuracy over the offline period, we ran a mixed-design ANOVA on mean accuracy scores (i.e., accuracy irrespective of the probabilities of events) with EPOCH (Epoch 4 vs Epoch 5) as a within-subject factor and AGE GROUP as a between-subject factor. The ANOVA revealed that mean accuracy scores significantly increased over the 24-hour delay (main effect of EPOCH: *F*(1, 246) = 13.55, *p* < 0.001, *η*_p_^2^ = 0.05). The amount of improvement was not uniform across the age groups (EPOCH x AGE GROUP interaction: *F*(8, 246) = 4.12, *p* < .001, *η*_p_^2^ = 0.12). Significant offline learning was only detectable in the 7-8-year-old group (*M_Epoch 4_* = 91.1%, *M_Epoch 5_* = 94.8%, *p* < .001) and in the 11-13-year-old group (*M_Epoch 4_* = 95.7%, *M_Epoch 5_* = 96.8%, *p* = .045). Mean accuracy scores were comparable over the delay in the other age groups (*p*s > .058). These results suggest that, in terms of accuracy scores, none of the age groups showed forgetting in general skills, therefore, offline learning in terms of reaction times cannot be explained by a decreased accuracy over the offline period.

**References**

1 Juhasz, D., Nemeth, D. & Janacsek, K. Is there more room to improve? The lifespan trajectory of procedural learning and its relationship to the between- and within-group differences in average response times. *PLoS One* **14**, e0215116, doi:10.1371/journal.pone.0215116 (2019).

2 Janacsek, K., Fiser, J. & Nemeth, D. The best time to acquire new skills: age-related differences in implicit sequence learning across the human lifespan. *Dev. Sci.* **15**, 496-505 (2012).

3 Pan, S. C. & Rickard, T. C. Sleep and motor learning: is there room for consolidation? *Psychol. Bull.* **141**, 812 (2015).

4 Howard, J. H., Jr. & Howard, D. V. Age differences in implicit learning of higher-order dependencies in serial patterns. *Psychol. Aging* **12**, 634-656 (1997).

5 Song, S., Howard, J. H., Jr. & Howard, D. V. Implicit probabilistic sequence learning is independent of explicit awareness. *Learn. Mem.* **14**, 167–176 (2007).

6 Song, S., Howard, J. H., Jr. & Howard, D. V. Sleep does not benefit probabilistic motor sequence learning. *J. Neurosci.* **27**, 12475-12483, doi:10.1523/jneurosci.2062-07.2007 (2007).
